# Beneficial Effects of Fisetin, a Senotherapeutic Compound, in Women’s Reproductive Health and Diseases: Evidence from In Vitro to Clinical Studies

**DOI:** 10.3390/nu18030393

**Published:** 2026-01-25

**Authors:** Samya El Sayed, D’leela Saiyed, Valeria I. Macri, Awurakua Asamoah-Mensah, James H. Segars, Md Soriful Islam

**Affiliations:** Department of Gynecology and Obstetrics, Division of Reproductive Sciences & Women’s Health Research, Johns Hopkins Medicine, Baltimore, MD 21205, USAjsegars2@jhmi.edu (J.H.S.)

**Keywords:** fisetin, senolytic properties, reproductive health, reproductive diseases, endometriosis, gynecologic cancers, uterine fibroids, PCOS, ovarian function, antioxidant properties

## Abstract

Fisetin is a naturally occurring flavonoid, a type of polyphenol found in fruits and vegetables such as strawberries, apples, persimmons, and onions. It has gained increasing attention for its antioxidant properties (enhancement of SOD1 and CAT activity and reduction of ROS), anti-inflammatory effects (suppression of NF-κB signaling), and senotherapeutic activity (senolytic and senomorphic effects). Although numerous studies have examined fisetin in the context of aging and chronic diseases, its role in women’s reproductive health has not been systematically explored. Mechanistically, fisetin regulates several pathophysiological processes, including ovarian aging, fibrosis, angiogenesis, and hormonal regulation, suggesting its potential relevance to female reproductive health and disease. Indeed, emerging evidence indicates that fisetin may support ovarian function and hormonal balance, modulate fibrosis and metabolism in benign gynecologic conditions, and suppress cell growth in gynecologic cancers. Early-phase clinical studies in non-gynecologic conditions suggest an acceptable safety profile, although evidence in reproductive health remains absent. This review summarizes current experimental and clinical evidence, identifies critical gaps in mechanistic understanding, and discusses future directions for advancing fisetin as a promising non-hormonal therapeutic option in reproductive health and diseases.

## 1. Introduction

Women’s reproductive health disorders, including endometriosis, uterine fibroids, polycystic ovary syndrome (PCOS), and gynecologic malignancies, are highly prevalent and contribute substantially to infertility, chronic pain, adverse pregnancy outcomes, and reduced quality of life worldwide [1,2,3,4,5]. In addition to their reproductive consequences, these conditions are frequently accompanied by metabolic, cardiovascular, and mental health comorbidities, underscoring the need for safe and effective long-term therapeutic strategies [6,7,8]. Current management heavily depends on hormonal therapies, surgery, and cytotoxic chemotherapy, which may be associated with significant side effects, limited durability of response, and unsuitability for women wishing to preserve fertility [9,10,11]. Consequently, there is growing interest in non-hormonal interventions that target shared pathways such as oxidative stress, chronic inflammation, fibrosis, metabolic dysregulation, and cellular senescence [12,13,14,15,16].

Fisetin is a dietary flavonol found in fruits and vegetables such as strawberries, apples, persimmons, and onions [17]. It exhibits pleiotropic biological activities, including antioxidant, anti-inflammatory, antifibrotic, and antitumor effects [18,19,20,21]. Importantly, fisetin has been identified as a senolytic agent capable of eliminating senescent cells and attenuating the senescence-associated secretory phenotype (SASP), thereby improving tissue function in preclinical models of aging and chronic disease [22,23]. These mechanisms are highly relevant to reproductive and gynecologic pathophysiology, where oxidative damage, extracellular matrix remodeling, mitochondrial dysfunction, altered immune responses, and cellular senescence may contribute to ovarian aging, subfertility, abnormal uterine bleeding, pelvic pain, and tumor growth [24,25,26,27].

A growing body of preclinical work suggests that fisetin is capable of modulating key pathogenic processes across multiple reproductive contexts, such as ovarian aging, fertility, menopause, endometriosis, uterine fibroids, PCOS, and gynecologic cancers [20,21,28,29,30]. Parallel early-phase clinical studies in non-gynecologic settings, for example, frailty, metabolic disease, and cancer survivorship, support the feasibility and emerging safety profile of fisetin in humans (NCT03675724; NCT05595499; NCT06113016). This review explores how fisetin may provide health benefits and reduce symptoms associated with women’s reproductive health, as well as benign and malignant gynecological conditions.

## 2. Methods

A comprehensive literature search was conducted using PubMed and Google Scholar up to December 2025. For studies related to gynecological diseases, the following search terms were used: “fisetin” AND (“uterine fibroids” OR “leiomyoma” OR “adenomyosis” OR “endometriosis” OR “polycystic ovary syndrome” OR “PCOS” OR “cervical cancer” OR “endometrial cancer” OR “ovarian cancer”). Only full-text articles published in English were included. Peer-reviewed original research articles providing mechanistic, in vitro, in vivo, or clinical evidence of fisetin’s effects in gynecological diseases were eligible for inclusion. Additionally, ClinicalTrials.gov was searched to identify all registered clinical trials involving fisetin across all conditions.

Studies were screened initially based on title and abstract, followed by full-text review of potentially relevant articles. For each preclinical study on gynecological diseases, data extracted included gynecological condition studied; study design (in vitro, in vivo); experimental model used; fisetin dosage and treatment regimen; and key findings across multiple features, including antitumor effects (cytotoxicity, proliferation, and apoptosis), histomorphological changes, hormonal and metabolic parameters, inflammatory and oxidative stress markers, anti-metastatic effects, and underlying molecular mechanisms (signaling pathways). For clinical trials, data extracted from ClinicalTrials.gov included trial identifier (NCT number), phase, target conditions, participant demographics (sex and age), enrollment numbers, fisetin dosing regimens, study objectives, study location, and current trial status.

As this article is intended as a narrative review, it does not follow a systematic review framework and therefore does not include formal quality assessment or risk-of-bias evaluation of individual studies. In addition, the literature search was restricted to English-language publications, which may have introduced language bias and resulted in the exclusion of relevant studies published in other languages.

## 3. Overview of Fisetin

**Figure 1:**
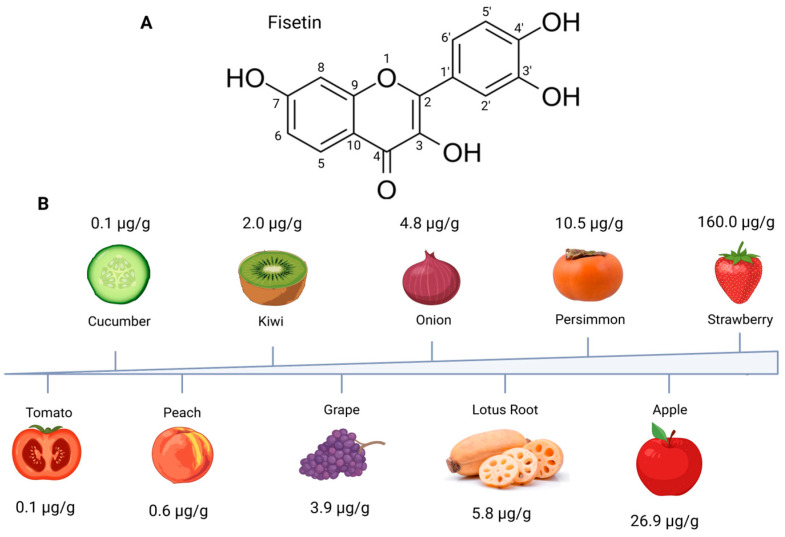
Dietary sources of fisetin and their relative concentrations. (**A**) Chemical structure of fisetin. (**B**) Fisetin content is expressed as µg per gram wet food. Figures made in BioRender.com.

The chemical formula of fisetin (3,3′,4′,7-tetrahydroxyflavone) was first described by the Austrian chemist Josef Herzig in 1891 [31], and its chemical structure was further elucidated by S. Kostanecki in the 1890s. The first chemical synthesis of fisetin was performed in 1904 [32]. Fisetin is distinguished by four hydroxyl (–OH) groups located at positions 3 and 7 on the A and C rings, and at positions 3′ and 4′ on the B ring (Figure 1). Its chemical structure, particularly the 3′,4′-dihydroxy group, allows it to neutralize reactive oxygen species (ROS) through hydrogen donation [32].

Fisetin is a widely distributed but relatively low-abundance flavonoid in the plant kingdom. High concentrations are found in fruits, vegetables, nuts, and certain medicinal plants. Among commonly consumed foods, strawberries are considered one of the richest natural sources of fisetin, containing approximately 160 μg/g fresh weight [17]. Apples, persimmons, grapes, onions, and lotus roots also contain measurable amounts, though typically at lower levels [17]. Lesser known but significant sources include peaches, kiwis, tomatoes, and cucumbers [17]. In addition to edible plants, fisetin is present in various traditional medicinal herbs, including *Rhus verniciflua* [33].

### 3.1. Pharmacokinetics of Fisetin

**Figure 2:**
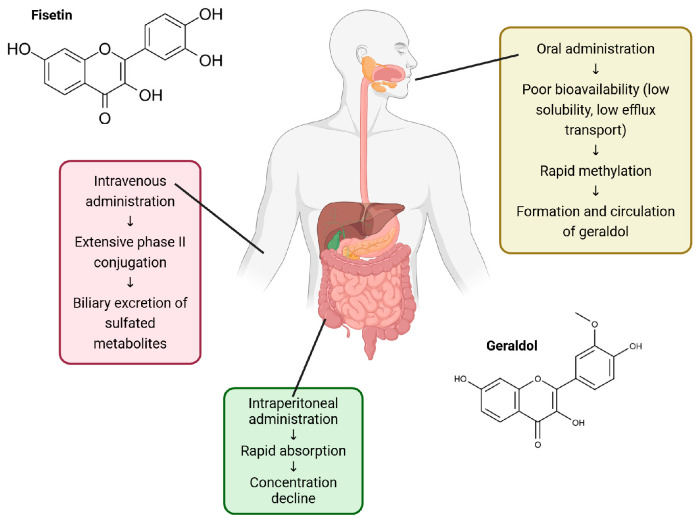
Pharmacokinetics of fisetin. Figures made in BioRender.com.

Fisetin undergoes rapid absorption followed by extensive conjugative metabolism and poor oral bioavailability driven by low solubility and efflux transport (Figure 2). After intravenous administration (30 mg/kg) in rats, fisetin undergoes extensive phase II conjugation, primarily to glucuronides and sulfates, with plasma area under the curve (AUC) ratios of parent–glucuronide–sulfate = 1:6:21 and biliary ratios of 1:4:75, indicating predominant biliary excretion of sulfated metabolites mediated by P-glycoprotein [34]. An earlier study by Zhang et al. identified 53 in vivo and 14 in vitro metabolites derived from oxidation, reduction, methylation, sulfation, and glucuronidation [35]. In mice, fisetin is rapidly O-methylated by catechol-O-methyltransferase to form geraldol, its major circulating metabolite. After oral administration (100–200 mg/kg), geraldol showed a higher Cmax and AUC than fisetin, while absolute bioavailability of fisetin remained low (7.8–31.7%), indicating rapid methylation and poor systemic exposure [36]. Following intraperitoneal administration at 223 mg/kg, plasma fisetin levels reached a peak concentration of 2.5 µg/mL within 15 min and declined in a biphasic manner, with an initial half-life of 0.09 h and a terminal half-life of 3.1 h. Notably, the major metabolite geraldol accumulated at higher concentrations than fisetin itself within Lewis lung tumors [37].

Formulation strategies have substantially improved fisetin pharmacokinetics. Liposomal fisetin enhanced bioavailability 47-fold and delayed tumor growth compared to free fisetin [38], while fisetin nanoemulsions achieved 24-fold greater systemic exposure and antitumor efficacy at lower doses [39]. Nanocochleates further increased relative bioavailability 141-fold and prolonged systemic circulation [40]. Polymeric and PLGA-based nanoparticles improved dissolution 3-fold and intestinal permeability 4.9-fold [41], and self-nanoemulsifying systems (SNEDDSs) produced 6–9-fold higher bioavailability with superior neuroprotection [42]. In healthy human individuals, a fenugreek–galactomannan hydrogel formulation (FF-20) increased fisetin’s AUC_0–12 h_ 26.9-fold and its Cmax 23-fold, while reducing methylation to geraldol and showing excellent tolerability [43].

Overall, fisetin exhibits rapid absorption but extensive metabolism and low bioavailability, which can be substantially improved by advanced delivery systems such as liposomes, nanoemulsions, nanocochleates, polymeric nanoparticles, SNEDDSs, and hydrogel formulations.

### 3.2. Antioxidant Properties of Fisetin

The antioxidant capacity of fisetin starts with the molecule itself, as the 3′,4′-dihydroxy groups on the B ring easily donate hydrogen atoms and act as the main sites that neutralize free radicals [44]. Using the oxidative stress-sensitive HT-22 neuronal cell model, Ishige et al. reported that fisetin can reduce ROS generation, maintain intracellular glutathione (GSH) levels, and prevent excess Ca^2+^ influx during glutamate-induced toxicity, demonstrating multiple protective modes in neurons under oxidative stress [45]. Fisetin also activated endogenous defense pathways by promoting Nrf2 nuclear translocation and ARE-driven HO-1 expression in human endothelial cells, an effect diminished by PKC-δ or p38 inhibition, which also reduces its protective action against H_2_O_2_-induced injury [46]. A complementary study with HepG2 cells showed that fisetin can stabilize Nrf2 protein post-transcriptionally by slowing ubiquitin–proteasome-mediated degradation, thereby increasing Nrf2 half-life and upregulating HO-1, GCLC, GCLM, and NQO1 [47]. Oral fisetin improved pancreatic antioxidant status and lowered lipid peroxidation while normalizing glycemia and inflammatory readouts in streptozotocin-diabetic rats [48]. After traumatic brain injury in mice, fisetin reduced malondialdehyde (MDA), restored glutathione peroxidase (GPx) activity, decreased neuronal apoptosis, and improved neurological function via Nrf2–ARE activation; notably, Nrf2 deletion abrogated these antioxidant benefits [49]. In cardiovascular stress models, fisetin lowered myocardial ROS, increased SOD1, CAT, and HO-1, and suppressed pro-hypertrophic MAPK and mTOR signaling [19].

**Figure 3:**
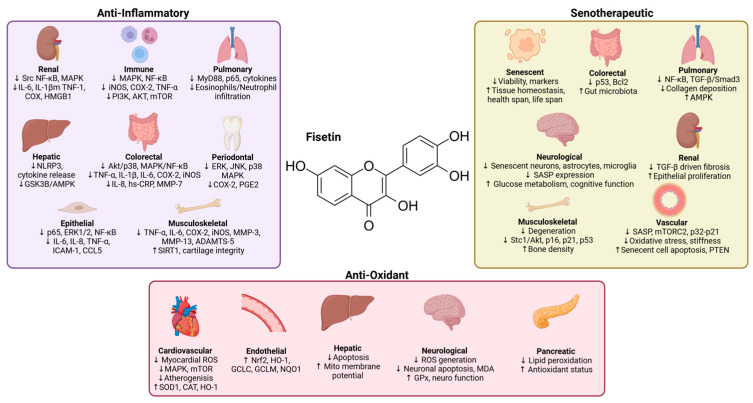
Biological effects of fisetin: anti-inflammatory, antioxidant, and senotherapeutic actions across organ systems. Figures made in BioRender.com.

Fisetin also showed radioprotective antioxidant actions. In γ-irradiated cells, fisetin reduced ROS generation, prevented lipid peroxidation and DNA/protein oxidation, and preserved mitochondrial membrane potential to limit apoptosis [50]. At the vascular–metabolic interface, fisetin dampens atherogenic pathways by inhibiting Cu^2+^-driven LDL oxidation and reducing macrophage oxLDL uptake through downregulation of PPARγ-dependent CD36 expression [51]. Importantly, these activities align with broader flavonoid SARs (structure–activity relationships), showing that o-dihydroxyls on ring B and low oxidation potentials track with high ferric-reducing power and radical-trapping capacity [44,52,53]. Overall, fisetin acts as a chemically efficient radical scavenger that also amplifies endogenous antioxidant defenses across neuronal, endothelial, hepatic, and cardiac contexts (Figure 3).

### 3.3. Anti-Inflammatory Properties of Fisetin

Extensive evidence from in vitro and in vivo studies demonstrated that fisetin effectively suppresses inflammation across diverse pathological conditions, including metabolic, respiratory, neurodegenerative, cardiovascular, renal, and musculoskeletal diseases (Figure 3). Its anti-inflammatory actions are largely mediated by the suppression of NF-κB, MAPK, PI3K/AKT/mTOR, and TLR4 signaling pathways.

In mast cells, fisetin effectively inhibited the activation of MAPK and NF-κB signaling, leading to reduced secretion of inflammatory mediators, including TNF-α, IL-1β, IL-4, IL-6, and IL-8 [18]. In macrophage and microglial models, fisetin was reported to downregulate iNOS, COX-2, and TNF-α, while inhibiting the nuclear translocation of NF-κB (p65) and phosphorylation of upstream kinases such as Src, Syk, and JNK [54,55,56]. Fisetin also suppressed PI3K, AKT, and mTOR phosphorylation, while it promoted autophagosome–lysosome fusion in lipopolysaccharide (LPS)-stimulated macrophages [57]. Gutiérrez-Venegas et al. [58] reported that fisetin can inhibit ERK, JNK, and p38 MAPK activation in human gingival fibroblasts, leading to decreased COX-2 expression and PGE_2_ release, supporting its anti-inflammatory action in periodontal inflammation. In epithelial tissues, fisetin suppressed cytokine-driven inflammation by attenuating NF-κB p65 nuclear translocation and ERK1/2 phosphorylation, as well as reducing IL-6, IL-8, TNF-α, ICAM-1, and CCL5 expression [59,60]. Similarly, in vascular endothelial cells, fisetin inhibited ROS-NF-κB signaling, CAM expression, and leukocyte adhesion, suggesting vascular protection against hyperglycemia-induced inflammation [61].

Animal studies further support the strong anti-inflammatory efficacy of fisetin in multiple pathologies. Sahu et al. [62] showed that fisetin can attenuate DSS-induced colitis by suppressing Akt/p38 MAPK/NF-κB signaling and reducing TNF-α, IL-1β, IL-6, COX-2, and iNOS expression. In allergic asthma and airway inflammation models, fisetin suppressed MyD88 and NF-κB (p65) activation; decreased infiltration of eosinophils and neutrophils; and reduced cytokines, including IL-4, IL-5, IL-13, IL-17, and IL-33 [63,64]. In LPS-induced septic acute kidney injury, fisetin improved renal function by inhibiting Src-mediated NF-κB and MAPK (p38, ERK1/2, and JNK) pathways, as well as suppression of IL-6, IL-1β, TNF-α, COX-2, and HMGB1 expression [65]. In hepatic ischemia–reperfusion injury, fisetin exerted protection through GSK3β/AMPK activation, which inhibited NLRP3 inflammasome components (caspase-1, IL-1β, and IL-18) and proinflammatory cytokine release [66].

In degenerative and metabolic diseases, fisetin modulates chronic inflammation via transcriptional and epigenetic mechanisms. Fisetin inhibited NF-κB activation and histone acetylation in hyperglycemia-exposed monocytes, and suppressed IL-6 and TNF-α release and CBP/p300 and histone acetyltransferase activity [67]. Combined with luteolin, fisetin synergistically reduced NF-κB, ROS, and HAT activity, while it upregulated SIRT1 and FOXO3a [68]. In osteoarthritis, fisetin also suppressed IL-1β-induced production of TNF-α, IL-6, COX-2, iNOS, MMP-3, MMP-13, and ADAMTS-5 via activation of SIRT1 [69,70].

Clinical and advanced pharmacological studies also support its anti-inflammatory potential. In colorectal cancer patients receiving chemotherapy, 100 mg/day fisetin reduced plasma IL-8, hs-CRP, and MMP-7 levels, indicating systemic anti-inflammatory potential [71]. Additionally, newly synthesized fisetin derivatives demonstrated stronger inhibition of NF-κB, inflammasome, and ER stress pathways with reduced cytotoxicity [72].

Overall, these findings strongly support fisetin as a potential anti-inflammatory flavonoid that suppresses key inflammatory mediators such as TNF-α, IL-1β, IL-6, COX-2, iNOS, NF-κB, and MAPK while regulating upstream kinases and epigenetic modulators.

### 3.4. Senotherapeutic Properties of Fisetin

Senescence is a cellular state characterized by cell cycle arrest even with a favorable microenvironment [73]. It is caused by increased activity of cyclin-dependent kinase inhibitors (p16 and p21), leading to resistance to apoptosis and metabolic changes, including accumulation of senescence-associated β-galactosidase (SA-β-gal) [73]. Senescent cells develop a senescence-associated secretory phenotype (SASP), which involves secretion of proinflammatory cytokines, chemokines, and proteases [74]. Persistent SASP leads to inflammation, disruption of tissue homeostasis, and local induction of senescence in neighboring cells [74]. Senescence may be induced by various noxious intracellular or extracellular stimuli, including DNA damage; oncogene activation; telomere dysfunction; and oxidative, metabolic, or mechanical stress [23]. SASP profiles are highly heterogeneous and depend on cell type; tissue microenvironment; and the nature, intensity, and duration of stress [75].

Pharmacological strategies targeting senescent cells are broadly referred to as senotherapeutics and can be divided into senolytic and senomorphic agents [76]. Senolytic drugs selectively eliminate senescent cells. On the other hand, senomorphic drugs modulate SASP and suppress secretory potential without eliminating senescent cells [76]. This section summarizes evidence supporting fisetin as both a senolytic and senomorphic agent across multiple disease conditions and organ systems.

#### 3.4.1. Senolytic Effects

The landmark study by Yousefzadeh et al. [77] reported the foundational role of fisetin as a natural senolytic agent capable of selectively eliminating senescent cells in aged and progeroid mice. Fisetin treatment reduced senescence markers across multiple tissues, restored tissue homeostasis, and extended both healthspan and lifespan. These findings demonstrated that fisetin treatment could reverse molecular hallmarks of aging through a “hit-and-run” mechanism. Consistent with this, Zhu et al. [22] reported that fisetin induced apoptosis specifically in senescent human endothelial cells while sparing proliferating cells, indicating its tissue-selective senolytic potential. Mechanistically, fisetin acts through multiple signaling pathways to regulate senescence. Ji et al. [78] reported that fisetin can ameliorate type 2 diabetes-related vascular aging by targeting the PI3K/Akt/Bcl-2/Bcl-xl axis, promoting apoptosis of senescent endothelial cells, suppressing SASP factors, and enhancing the therapeutic efficacy of metformin, though direct dose equivalence and mechanistic comparisons were not established. Mahoney et al. [79] extended these findings, showing that fisetin decreased vascular senescence by reducing the viability of senescent endothelial cells while sparing nonsenescent cells. Fisetin also reduced oxidative stress and inflammation in aged mice, improved nitric oxide bioavailability, and reduced arterial stiffness.

Beyond vascular systems, fisetin exhibits strong antifibrotic effects. In lupus nephritis, Ijima et al. [80] revealed that fisetin selectively reduced senescent tubular epithelial cells and myofibroblasts, suppressing TGF-β-driven fibrosis and restoring renal epithelial proliferation. In idiopathic pulmonary fibrosis models, Zhang et al. [81] demonstrated that fisetin activated AMPK and inhibited NF-κB and TGF-β/Smad3 signaling, reducing alveolar epithelial senescence, collagen deposition, and fibroblast transdifferentiation. Fisetin also exhibits neuroprotective and systemic senolytic actions. Huard et al. [82] demonstrated that fisetin reduced senescent neurons, astrocytes, and microglia in aged sheep; downregulated senescence and inflammasome genes in peripheral organs; and improved brain and systemic aging markers. Fisetin’s benefits extend to musculoskeletal and degenerative disorders as well. Hambright et al. [83] demonstrated that fisetin significantly reduced senescent cell burden in murine chondrocytes (ATDC5) and pre-osteoblasts (MC3T3) in vitro, while Zhao et al. [84] demonstrated reversal of premature aging in telomerase-deficient mice via inhibition of the Stc1/Akt signaling pathway, promoting apoptosis of senescent cells and reduction of p16INK4a/p21CIP1 expression.

Furthermore, fisetin exhibits antitumor and adjuvant potential in cancer therapy. Russo et al. [85] showed that fisetin enhanced the radiosensitivity of resistant cancer cells through AMPK activation and ERK inhibition, promoting autophagy, apoptosis, and attenuation of senescence-associated inflammation. The combination of radiation and fisetin reduced SA-β-gal activity by 40–50% and decreased senescence markers (p16, p21), while inducing both apoptotic and autophagic cell death [85]. Notably, the translational relevance of fisetin has been validated in higher-order species. Colman et al. [86] demonstrated that combined dasatinib and fisetin treatment in aged rhesus monkeys significantly reduced epidermal p16^+^ and p21^+^ senescent cells without adverse effects, confirming the safety and efficacy of combination senolytic therapy in primates.

#### 3.4.2. Senomorphic Effects

In addition to senolytic activity, several studies demonstrate fisetin’s ability to suppress SASP. Ji et al. [78] reported that fisetin can ameliorate type 2 diabetes-related vascular aging by suppressing SASP factors, while Mahoney et al. [79] showed that fisetin decreased vascular senescence, oxidative stress, and inflammation in aged mice. Fisetin also demonstrated senomorphic activity in fibrotic conditions. In idiopathic pulmonary fibrosis models, Zhang et al. [81] demonstrated that fisetin activated AMPK and inhibited NF-κB and TGF-β/Smad3 signaling, reducing alveolar epithelial senescence, collagen deposition, and fibroblast transdifferentiation. Similarly, Ashiqueali et al. [87] reported that fisetin alleviated DSS-induced colitis by downregulating p53, Bcl2, and proinflammatory mediators and restoring beneficial gut microbiota such as Akkermansia muciniphila, indicating senescence- and inflammation-targeted modulation of intestinal homeostasis. In osteoarthritic models, Jacob et al. [88] found that fisetin and resveratrol reduced senescence in chondrogenic progenitor cells by downregulating p53 and SASP mediators and suppressing inflammation and matrix degradation. Liposomal fisetin formulations, as demonstrated by Henschke et al. [89], enhanced senomorphic efficacy by reducing IL-6 and IL-8 secretion without inducing cytotoxicity.

#### 3.4.3. Preventive and Anti-Aging Effects

Kim et al. [90] found that fisetin delays vascular aging by upregulating PTEN and inhibiting mTORC2-Akt(Ser473) signaling, as well as reduced p53-p21 activation and senescence phenotypes in vascular smooth muscle cells. Similarly, Hambright et al. [83] reported that fisetin preserved bone density and alleviated frailty-related skeletal degeneration in Zmpste24^−^/^−^ progeria mice when administered before advanced pathology developed. Beyond these protective effects, Fang et al. [91] found that early-life fisetin administration enhanced glucose metabolism, cognitive function, and reduced SASP expression in male mice, indicating sex-dependent responses to preventive interventions.

Overall, these studies support fisetin as a broad-spectrum senotherapeutic agent that targets multiple aging pathways and disease conditions from metabolic and cardiovascular dysfunctions to fibrosis, neurodegeneration, inflammation, and cancers (Figure 3). By modulating key molecular targets, including PI3K/Akt, PTEN/mTORC2, AMPK/NF-κB, and TGF-β/Smad3, fisetin effectively clears senescent cells, suppresses SASP, and restores tissue regeneration and homeostasis.

## 4. Role of Fisetin in Women’s Reproductive Health

### 4.1. Ovarian Aging

Women are born with a finite number of oocytes stored within primordial follicles. As women age, chromosomal, genetic, mitochondrial, and cytoplasmic factors progressively impair both the quality and quantity of oocytes [92]. This process, known as ovarian aging, results from the lifelong depletion of the primordial follicle pool and affects all women throughout their reproductive years [92]. By the mid-30s, the rate of follicle depletion accelerates, contributing to infertility and increased risk of adverse obstetric outcomes [93]. Ovarian aging is further characterized by heightened oxidative stress, inflammation, mitochondrial dysfunction, and accumulation of senescent cells [24].

At the molecular level, gene variants in Forkhead Box O3 (FOXO3) and Klotho have been implicated in ovarian aging [25]. Both genes play key roles in cellular protection by regulating oxidative stress responses and promoting longevity-associated pathways [94]. Declines in estrogen and progesterone with age also contribute by disrupting menstrual cycle regulation and follicular development [94]. Current therapeutic approaches for ovarian aging include antioxidant supplementation, stem cell-based therapies, and hormonal or growth factor support [95]. Since many of these interventions aim to reduce cellular stress, fisetin’s antioxidative and senolytic properties offer potential therapeutic value [23]. Fisetin reduces oxidative stress through PTEN-mediated inhibition of the pro-oxidant enzyme NADPH oxidase 1 (NOX1), which neutralizes ROS. Its senolytic activity is also significant, as clearance of senescent cells may alleviate inflammation and restore tissue function [23].

**Figure 4:**
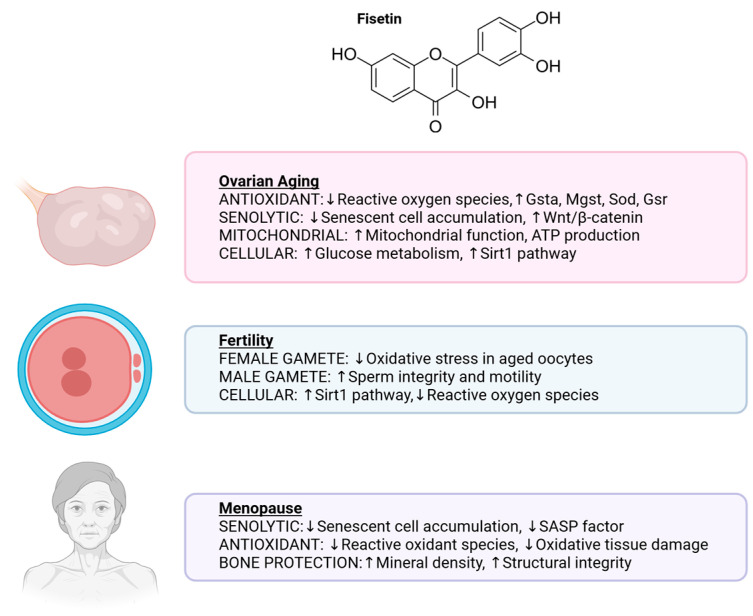
Roles of fisetin in ovarian aging, fertility, and menopause. Figures made in BioRender.com.

Several animal studies have been conducted to test the potential benefits of fisetin for ovarian aging (Figure 4). In Hyline White laying chickens, Yang et al. [96] demonstrated that 50 mg/kg/day of fisetin enhanced antioxidant defense, improved egg production, and increased overall egg quality without signs of toxicity. Expression of antioxidant genes, including Gsta, Mgst, Sod, and Gsr, was upregulated, while Western blot analyses showed enhanced glucose metabolism in aged chickens receiving fisetin [96]. In another study using the same chicken strain, Dong et al. [97] examined granulosa cell function by treating cells with fisetin at concentrations of 0–80 µM. The activation of the Nrf2/HO-1 pathway and upregulation of the Wnt/β-catenin signaling pathway by fisetin was observed, which was associated with reduced expression of senescence-associated genes such as p53, p21, and p16 [97].

Using a murine model, Xing et al. [98] demonstrated that fisetin delays postovulatory oocyte aging through modulation of the Sirt1 pathway, a key regulator of mitochondrial function, apoptosis, and ROS accumulation. In vitro treatment with fisetin (1–20 μM) significantly reduced oxidative stress in aged oocytes and improved mitochondrial function, as evidenced by increased ATP content (0.49 ± 0.014 pmol vs. 0.43 ± 0.014 pmol, *p* < 0.05) [98]. However, contrasting findings were reported in reproductive-age mice treated with 5 mg/kg dasatinib plus 50 mg/kg quercetin compared with 100 mg/kg fisetin. While fisetin reduced senescence markers, it did not improve ovarian reserve or fertility outcomes [99].

Overall, these studies suggest the potential effects of fisetin to modulate pathways involved in ovarian aging, while also highlighting the need for further research.

### 4.2. Fertility

Fertility refers to an individual’s ability to conceive and is regulated in women through a monthly reproductive cycle [100]. In women, a 5-day fertile window includes the days leading up to ovulation and ovulation day itself, as sperm can reside in the female reproductive tract for many days [101]. Numerous factors influence fertility, including age, sexually transmitted infections, lifestyle choices, body weight, and environmental exposures [102]. Among these determinants, oocyte quality is one of the strongest predictors of successful embryo development. Postovulatory oocyte aging, in particular, is associated with reduced fertilization potential and impaired embryonic development [98].

As described in the previous section, Xing et al. [98] demonstrated that fisetin protected aging mouse oocytes from oxidative stress and improved mitochondrial function by modulating the Sirt1 pathway. The Sirt1 pathway plays a critical role in reproductive function by protecting gametes from oxidative damage, regulating cellular energy and motility, controlling inflammation, and maintaining proper hormone signaling [103]. Although Sirt1 activity declines with age, fisetin’s modulation of this pathway was associated with enhanced mitochondrial ATP production and reduced oxidative stress in aged oocytes [98]. These findings suggest that fisetin might partially compensate for age-related Sirt1 decline through multiple mechanisms which support oocyte health, offering a potential strategy for fertility preservation.

In addition to its effects on female gametes, fisetin may benefit male fertility as well. A clinical study conducted in Iran evaluated whether fisetin supplementation could improve sperm integrity during cryopreservation [104]. In this study, 20 semen samples were divided into three groups: fresh non-frozen control, standard cryopreservation medium, and cryopreservation medium supplemented with 50 μM fisetin. Using sperm DNA fragmentation analysis and multiple chromatin integrity assays, Ezati et al. [104] found that fisetin significantly improved sperm DNA preservation and motility following cryopreservation. These results highlight the antioxidant capacity of fisetin and suggest that it may enhance assisted reproductive technologies by protecting sperm from cryo-induced oxidative damage.

Collectively, the available evidence, though limited, indicates that fisetin may support both female and male fertility by reducing oxidative stress, maintaining mitochondrial function, and preserving gamete integrity (Figure 4).

### 4.3. Menopause

Menopause is a natural transition in a woman’s life marked by the cessation of menses [105]. Clinically, menopause is defined as the absence of menstrual bleeding or spotting for 12 consecutive months and typically occurs between ages 45 and 55 [105]. The symptoms of menopause vary widely among individuals and may include weight gain, cognitive impairment, hot flashes, sleep disturbances, vaginal atrophy, decreased libido, and fatigue [105,106]. The loss of estrogen can increase the risk of osteoporosis due to a loss of bone density and raise cholesterol levels, which increase the rate of heart disease and stroke in women [107,108]. As menopause progresses, senescent cells accumulate in multiple tissues, including metabolic organs, bone, and components of the immune system [109]. These senescent cells secrete SASP factors, which promote inflammation, tissue dysfunction, and systemic decline [26]. Current therapies for menopausal symptoms primarily involve hormonal treatments aimed at relieving vasomotor symptoms, along with lifestyle-based interventions such as Pilates, which has been shown to improve pain and functional outcomes [110,111].

Given its senolytic and antioxidant properties, fisetin has emerged as a potential therapeutic candidate for reducing menopause-associated dysfunction (Figure 4). Fisetin can eliminate senescent cells that contribute to tissue dysfunction and inflammation and reduce oxidative stress caused by ROS [112]. Supporting this possibility, Hambright et al. [83] evaluated the effects of fisetin in murine chondrocyte (ATDC5) and pre-osteoblast (MC3T3) cell lines treated with 50 µM fisetin to investigate its role in bone health. Using Hounsfield unit measurements, bone mineral density assessments, and analyses of specific bone surfaces, they found that fisetin supported bone preservation and might help prevent bone loss, an essential consideration in postmenopausal osteoporosis [83].

While current evidence suggests that fisetin may alleviate key biological features of menopause, including oxidative stress, inflammation, senescence, and bone degeneration, additional studies are needed to expand this area of research.

## 5. Role of Fisetin in Benign Gynecological Diseases

### 5.1. Endometriosis

Endometriosis is a complex chronic inflammatory condition characterized by the presence of estrogen-dependent endometrial tissue (including both stroma and glands) outside the uterine lining [1]. It is estimated to affect between 6 and 10% of reproductive-aged females globally [113]. However, the true prevalence may be higher due to significant diagnostic delays, with an average time between symptom onset and diagnosis of 6.8 years [114]. Endometriosis commonly leads to chronic pelvic pain, heavy and painful menstrual bleeding (dysmenorrhea), pain during intercourse (dyspareunia), and pain during defecation (dyschezia), and it is a leading cause of infertility [115,116]. The condition is associated with a substantial mental health burden, including increased risk of depressive and anxiety symptoms, and adversely affects health-related quality of life [117]. Current treatment options include hormonal suppression (estrogen-progestin contraceptives or progestins) and surgical excision of endometriotic lesions or hysterectomy when childbearing is no longer desired. Unfortunately, many patients do not experience relief with these interventions; specifically, up to 34% experience recurrent pelvic pain within 12 months after discontinuation of hormonal medications, and 25% of those who undergo hysterectomy will experience recurrent pelvic pain [116]. Thus, there is an urgent need to identify novel pharmacological agents that can effectively alleviate symptoms, improve reproductive outcomes, and enhance quality of life for patients with endometriosis.

**Figure 5:**
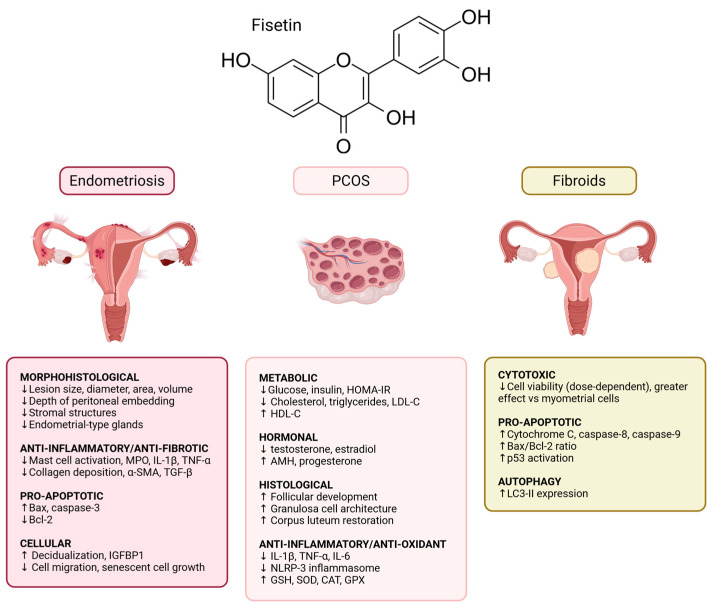
Therapeutic potential of fisetin in gynecological disorders: endometriosis, PCOS, and uterine fibroids. Figures made in BioRender.com.

Preclinical evidence suggests that fisetin may offer therapeutic benefit for endometriosis (Figure 5 and Table 1). Arangia and Marino et al. [20] induced endometriosis in Sprague Dawley rats using intraperitoneal injection of uterine tissue fragments and subsequently treated the animals with fisetin (40 mg/kg). Fisetin significantly altered the morphological, histological, inflammatory, and fibrotic characteristics of endometriotic lesions. Lesions from fisetin-treated rats were visibly smaller; less invasive; and demonstrated significantly reduced diameter, area, and volume [20]. Histologically, fisetin reduced stromal density and the presence of endometrial-type glands. Fisetin also suppressed inflammation by decreasing mast cell activation, lowering MPO activity, and reducing IL-1β and TNF-α expression [20]. Antifibrotic effects were evident through reduced collagen deposition on Masson trichrome staining and decreased α-SMA and TGF-β expression. Additionally, fisetin promoted apoptosis, as reflected by increased TUNEL-positive cells, increased Bax and caspase-3 expression, and reduced Bcl-2 levels on Western blots [20].

Defective decidualization is a central feature of endometriosis-related infertility [118] and recurrent pregnancy loss [119]. Single-cell RNA sequencing of menstrual effluent from patients with endometriosis has shown a higher proportion of endometrial stromal cells with proinflammatory and senescent-like phenotypes compared to controls [120]. Delenko et al. [121] evaluated the effects of fisetin on primary human endometrial stromal cells isolated from patients at a tertiary care center. Fisetin (25 μM or 50 μM) significantly enhanced decidualization, measured by IGFBP1 protein levels, without inducing cytotoxicity. It also reduced stromal cell migration at 25 μM in wound closure assays and decreased senescent cell burden, as assessed by reduced lipofuscin accumulation [121]. Mechanistically, fisetin downregulated phosphorylation of AKT, PRAS40, ERK1, and ERK2 [121], suggesting inhibition of pro-survival and pro-migratory signaling pathways.

Overall, these studies indicate that fisetin may target multiple pathological features of endometriosis, including inflammation, fibrosis, senescence, impaired decidualization, and aberrant cellular signaling (Figure 5 and Table 1), and warrant additional studies to determine its efficacy and safety in humans.

**Table 1:** Effects of fisetin treatment on benign and malignant gynecological diseases.

Condition	Study (Year)	Biological Model	Fisetin Doses Used	Key Findings
ENDOMETRIOSIS	Arangia et al. [20]	Female Sprague Dawley rats (250 g) with induced endometriosis (intraperitoneal injection of uterine fragments)	40 mg/kg for 7 days (oral gavage)	Morphological: No change in cyst number; ↓ lesion size, diameter, area, volume; ↓ depth of peritoneal embedding Histological: ↓ stromal structures, endometrial-type glands Inflammatory: ↓ mast cell activation (reduced chymase and tryptase staining), ↓ NLRP3, ASC, cleaved caspase-1, ↓ NF-κB, ↓ IL-1β, TNF-α Oxidative stress: ↓ PAR positive expression, ↓ nitrotyrosine expression, ↓ MDA levels Fibrotic: ↓ collagen, α-SMA, TGF-β Apoptotic: ↑ TUNEL+ cells, Bax, caspase-3; ↓ Bcl-2
	Delenko et al. [121]	Primary culture of human endometrial stromal cells isolated from menstrual effluent	0, 25 μM, 50 μM	Decidualization: ↑ IGFBP1 protein levels Migration: ↓ cell migration at 25 μM (wound closure assay) Senescence: ↓ NanoJaggs accumulation Safety: Non-toxic at tested concentrations Molecular: ↓ AKT, PRAS40, ERK1, ERK2 phosphorylation on Western blots
UTERINE FIBROIDS	Lee et al. [29]	Primary culture of human leiomyoma and myometrial cells from patients undergoing hysterectomy	0, 5, 10, 20, 40, 60, 80, 100 μM	Cytotoxicity: Dose-dependent reduction in leiomyoma and myometrial cell viability as seen on MTT assay Selectivity: Greater fold change in apoptosis in leiomyoma vs. myometrial cells starting at 20 μM In leiomyoma cells on Western blots: 1. Intrinsic and extrinsic apoptosis: Bcl-2, Bax, cytochrome c, Apaf-1, caspase-3, caspase-6, caspase-8, caspase-9, PARP 2. p53-mediated pathway: p-p53, p-Cyclin B1 3. MAPK pathway: p-p38, p-ERK, p-JNK 4. Autophagy: Beclin-1, Atg7, LC3-I, LC3-II, total mTOR, Akt, p-mTOR, p-Akt
POLYCYSTIC OVARY SYNDROME (PCOS)	Moustafa et al. [122]	Wistar adult female rats with letrozole-induced PCOS (1 mg/kg/day for 21 days)	1.25 mg/kg, 2.5 mg/kg oral administration for 14 days following PCOS induction	Metabolic: ↓ serum total cholesterol, serum insulin, serum glucose, HOMA-IR Hormonal: ↓ LH, FSH; ↑ AMH Histological: Normalized follicular development, granulosa cell architecture, presence of corpus luteum restoration Anti-inflammatory: ↓ ovarian IL-1β, NF-κBp65 Antioxidant: ↓ Nrf2 gene expression
	Chahal et al. [123]	Sprague Dawley rats (9–12 weeks) with mifepristone-induced PCOS (20 mg/kg/day orally for 13 days)	20 mg/kg (low dose), 40 mg/kg (high dose) fisetin PO after induction for 21 days	Anthropometric: ↓ body weight Metabolic: ↓ fasting blood glucose, fasting insulin, HOMA-IR Hormonal: ↓ testosterone, estradiol, LH; ↑ progesterone, FSH (dose-dependent) Histopathological: ↓ count and size of cyst follicles; healthy developing follicles with oocyte and well-defined granulosa cells Anti-inflammatory: ↓ TNF-α, IL-6 Antioxidant: ↑ GSH, SOD
	Mihanfar et al. [28]	Wistar female rats (42-day-old) with letrozole-induced PCOS (1 mg/kg orally for 21 days)	10 mg/kg oral dose after induction for one month	Anthropometric: ↓ final body weight, ovary weight Histomorphological: ↓ number of cystic follicles, restoration of corpus luteum Hormonal: ↓ testosterone; ↑ estradiol, progesterone Metabolic: ↓ serum fasting glucose, HOMA-IR, cholesterol, triglyceride, LDL-C, HDL-C Antioxidant: ↑ CAT, SOD, GPX Molecular: ↑ SIRT1 mRNA levels, *p*-AMPK protein expression, ovarian SIRT1 protein expression; ↓ CYP17A1 mRNA levels, CYP17A1 protein expression
OVARIAN CANCER	Liu et al. [30]	Human ovarian cancer cell lines A2780 and OVCAR-3	0, 25, 50, 100 μM	Cytotoxicity: ↓ cell proliferation (dose-dependent), ↓ proportion of viable cells (MTT assay, AV/PI staining followed by flow cytometry) Apoptosis: ↓ cytochrome C mitochondrial RNA Necroptosis: ↑ ZBP1, RIP3, MLKL protein expression (when combined with z-VAD pan-caspase inhibitor)
	Carmi et al. [124]	Human ovarian cancer cells (A2780) co-cultured with HS-5 to confer platinum resistance	10 μM	Platinum resistance: Restored sensitivity to platinum prodrug RJY13 as evidenced by cleaved PARP protein in A2780 cells Molecular: ↑ ERK1/2 phosphorylation and activation
	Jafarzadeh et al. [125]	Human ovarian cancer cell line A2780	0, 50, 75, 100 μg/mL (combined with cisplatin 0.1 or 0.5 μg/mL)	Synergy: Enhanced cisplatin efficacy at all dose combinations on MTT assay; ↓ proportion of viable cells even when cisplatin used below its IC50 of 0.75 μg/mL
	Xiao et al. [126]	In vitro: SKOV3 human ovarian cancer cell line In vivo: SKOV3 xenograft mouse model in BALB/c athymic nude mice	In vitro: 10, 30, 100, 300 μmol/L (both fisetin and fisetin micelles) In vivo: 50, 100 mg/kg (both fisetin and fisetin micelles) intraperitoneal injection for 4 weeks, 4 consecutive days per week	In vitro: Superior cytotoxicity and enhanced apoptosis induction with polymeric micelle encapsulation; fisetin IC50: 61.2 μM, TGI: 34.8%; fisetin micelles IC50: 48.2 μM, TGI: 48.7% In vivo: Fisetin (50 mg/kg) led to 53.6% tumor growth inhibition; fisetin micelles reached 70.7% inhibition after 21 days; ↓ tumor size and weight
CERVICAL CANCER	Afroze et al. [21]	In vitro: HeLa human cervical cancer cell line	In vitro: 0, 50 μM for 48 h	Proliferation: ↓ cellular proliferation in dose- and time-dependent manner Apoptosis: Induced via intrinsic (↑ BAX, BAK1, caspase-9, APAF1; ↓ BCL-2) and extrinsic pathways (↑ FAS, FASL, TNF-family ligands, caspase-8) Anti-inflammatory: ↓ proinflammatory cytokines (IL-1 family, IL-4, IL-11) and chemokines (MCP-1, MIP-1β) Molecular: ↓ MAPK and PI3K/AKT/mTOR pathways; ↑ ATM, ATF2, VHL, and p53 activation
	Ying et al. [127]	In vitro: HeLa human cervical cancer cell line In vivo: HeLa xenograft in immunodeficient nude mice (BALB/c nu/nu male mice, 5–6 weeks old, 18–22 g)	In vitro: 0, 20, 40, 80 μM In vivo: 2 mg/kg or 4 mg/kg body weight intraperitoneal injection twice weekly for 35 days	Cytotoxicity: Dose-dependently and time-dependently ↓ cellular viability; IC_50_ of 52 ± 0.9 μM (24 h), 36 ± 0.5 μM (48 h) Molecular: Sustained activation of ERK1/2 phosphorylation mediating fisetin-induced apoptosis In vivo: ↓ growth rate of tumors compared with control group (*p* < 0.05), with inhibition rates of 82.65% and 92.62% for 2 mg/kg and 4 mg/kg
	Chou et al. [128]	Human cervical adenocarcinoma SiHa and CaSki cells	0, 10, 20, 40 μM for 48 h	Anti-metastatic: ↓ motility and invasiveness in concentration-dependent manner at non-toxic concentrations at 20 and 40 μM Molecular: Dephosphorylation of p38 MAPK, disruption of NF-κB nuclear translocation, repression of uPA gene expression
	Lin et al. [129]	In vitro: HeLa human cervical cancer cell line In vivo: HeLa xenografts in BALB/c (5-week-old) female mice	In vitro: 40 μM fisetin + sorafenib (2.5 or 5 μM) In vivo: 4 mg/kg fisetin and 10 mg/kg sorafenib orally two times per week	Synergistic effect: Combined treatment significantly ↓ cell viability compared to each agent alone (*p* < 0.01) In vivo: ↓ subcutaneous tumor volume in combination group vs. other groups (*p* < 0.01) Molecular: Combined treatment upregulated DR5, activation of caspase-8 and caspase-3, ↑ Bax/Bcl-2 ratio

### 5.2. Uterine Fibroids

Uterine fibroids, or leiomyomas, are the most common benign tumors of the uterus and are present in up to 70% of reproductive-age women worldwide [2]. Common risk factors include increasing age, Black race, obesity, hypertension, and vitamin D deficiency [130]. Histologically, fibroids are monoclonal growths arising from cells in the myometrium (smooth muscle of the uterus). In addition to smooth muscle cells, fibroids contain several populations of immune cells [131] and fibroblasts that secrete a dense and stiff extracellular matrix [27]. Morphologically, they vary in size, number, and location, with subtypes including submucosal (abutting the endometrial lining), intramural (situated within the muscular wall of the uterus), and subserosal (developing on the outer surface of the uterus) subtypes [132]. Clinically, symptoms are present in 25–50% of patients and include heavy, irregular, or prolonged menstrual bleeding, painful periods, pelvic pressure, and abdominal bloating [133]. Treatment options include medical management, which is often inadequate, leaving patients to seek more invasive yet definitive surgical options such as myomectomy or hysterectomy [9]. Similarly to endometriosis, there is a pressing need to develop safe and effective treatment options for uterine fibroids.

To date, only one study has directly evaluated fisetin as a potential therapeutic for uterine fibroids [29] (Figure 5 and Table 1). In this study, fisetin decreased the viability of both leiomyoma and myometrial cells in a dose-dependent manner as measured by MTT assay. In leiomyoma cells, there was a statistical decline in viability starting at low concentrations, with decreasing viability at higher concentrations (20, 40, 60, 80, and 100 μM) [29]. In contrast, myometrial cells showed no significant changes at the lowest fisetin concentration (10 μM), but significance was evident starting at 20 μM and increased in a dose-dependent manner [29]. Importantly, while both cell types exhibited higher rates of apoptosis when treated with fisetin, leiomyoma cells demonstrated significantly greater fold changes in apoptosis compared to myometrial cells starting at 20 μM [29]. Mechanistic studies indicated that fisetin activates multiple apoptotic pathways in leiomyoma cells. These include intrinsic and extrinsic apoptosis, MAPK- and p53-mediated signaling, and autophagy-related cell death [29]. This was evidenced by increased cytochrome C, caspase-8 and caspase-9, and Bax/Bcl-2 expression ratio, as well as activation of p53 and increased microtubule-associated protein 1A/1B-light chain 3-II (LC3-II) expression [29]. Overall, these findings suggest that fisetin promotes a multifaceted cell death response, with greater sensitivity observed in leiomyoma cells than in normal myometrial cells. Although limited, these early findings highlight the potential of fisetin as a non-hormonal therapeutic strategy for uterine fibroids.

### 5.3. Polycystic Ovary Syndrome (PCOS)

PCOS is a chronic, multisystemic syndrome with reproductive, metabolic, endocrine, and inflammatory features [134]. PCOS is a clinical diagnosis, and the most widely used criteria to diagnose PCOS are the Rotterdam criteria [11]. These criteria state that two out of three of the following need to be fulfilled for diagnosis: oligo- or anovulation (which typically manifests clinically as irregular or absent periods), clinical and/or biochemical signs of hyperandrogenism (clinical signs typically manifest as hirsutism, while biochemical signs include elevated free testosterone levels), and polycystic appearance of ovaries upon imaging (objectively defined as >12 follicles or ovarian volume >10 mL) [11]. It is the most common endocrine disorder in women of reproductive age, with a global prevalence of 9.2% [135].

Aside from the hallmark characteristics of PCOS, it is associated with reproductive and obstetric complications. These include increased rates of infertility, endometrial cancer, preeclampsia, and gestational diabetes [3]. It also increases the risk of cardiovascular and metabolic complications such as hypertension, dyslipidemia, obesity, and type 2 diabetes mellitus [6]. Additionally, PCOS is associated with increased rates of mental health conditions such as depression and anxiety [7]. Treatment of this condition includes a multi-faceted approach of lifestyle interventions managed according to manifesting symptoms. Lifestyle interventions are a mainstay of treatment: dietary changes and physical activity have shown benefit in improving the metabolic health of PCOS patients and decreasing the risk of long-term metabolic and cardiovascular complications [136]. Pharmacological options include metformin and hormonal birth control pills for menstrual regulation and hirsutism, with the option to add anti-androgens if hirsutism is not controlled [137]. For anovulatory-related infertility, options include clomiphene citrate or letrozole [138]. Three preclinical studies have examined the therapeutic potential of fisetin in PCOS, focusing on biochemical, hormonal, histological, inflammatory, and metabolic parameters (Figure 5 and Table 1).

In a letrozole-induced PCOS model in Wistar rats, Moustafa et al. [122] administered fisetin (1.25 mg/kg or 2.5 mg/kg) for 14 days following 21 days of letrozole treatment. Fisetin significantly decreased serum total cholesterol, insulin, glucose, and homeostatic model assessment for insulin resistance (HOMA-IR) compared with untreated PCOS rats. Although these metabolic markers did not fully return to control levels (except serum insulin at 2.5 mg/kg), the improvements were substantial [122]. Letrozole increased LH and FSH while decreasing AMH; fisetin reversed these abnormalities by reducing LH by 59% and FSH by 50%, and increasing AMH up to 400% at the higher dose [122]. Histologically, 2.5 mg/kg fisetin restored normal follicular development, improved granulosa cell architecture, and reestablished corpus luteum formation. Fisetin also reduced ovarian IL-1β levels to control values and significantly suppressed NLRP3 inflammasome expression in a dose-dependent manner [122].

Chahal et al. [123] induced PCOS in Sprague Dawley rats using mifepristone (20 mg/kg/day for 13 days) and subsequently treated the animals with low-dose (20 mg/kg) or high-dose (40 mg/kg) fisetin. Fisetin significantly reduced fasting glucose, fasting insulin, and HOMA-IR compared with PCOS controls. Hormonal profiles improved markedly, with reductions in testosterone, estradiol, and LH, and increases in progesterone and FSH toward normal values. Fisetin also attenuated inflammation by lowering TNF-α and IL-6 levels, while enhancing antioxidant defense through increased GSH and superoxide dismutase (SOD) [123]. These findings are consistent with earlier work by Mihanfar et al., who showed that fisetin normalized sex hormone levels (testosterone, estradiol, and progesterone) in letrozole-induced PCOS rats [28]. Fisetin improved fasting glucose, HOMA-IR, cholesterol, triglycerides, LDL-C, and HDL-C, and boosted antioxidant enzyme activity, including catalase (CAT), SOD, and GPX [28]. Importantly, fisetin demonstrated efficacy comparable to metformin, a first-line pharmacologic agent in PCOS management [28], though direct dose equivalence and mechanistic comparisons were not established.

Overall, these studies suggest that fisetin exerts broad therapeutic effects in PCOS by improving metabolic dysfunction, restoring hormonal balance, reducing inflammation, enhancing antioxidant capacity, and normalizing ovarian morphology (Figure 5 and Table 1). While promising, these findings are limited to animal studies, and clinical research is needed to determine whether fisetin may serve as a safe, effective, and non-hormonal therapeutic option for women with PCOS.

## 6. Role of Fisetin in Malignant Gynecological Diseases

### 6.1. Ovarian Cancer

Ovarian cancer refers to an array of tumors that originate in the ovaries or fallopian tubes. It is associated with significant morbidity and mortality, leading to more deaths than any other gynecological cancer in the United States [4]. Several risk factors contribute to its development, including genetic predispositions such as germline pathogenic variants in BRCA1/BRCA2 or Lynch syndrome, as well as smoking, endometriosis, infertility, and postmenopausal estrogen replacement therapy [139,140]. The majority of patients (about 80%) are diagnosed at advanced disease stage [139]. Currently, the mainstay of treatment relies on surgical resection and platinum-based chemotherapy [139]. Despite these interventions, the five-year relative survival rate between 2015 and 2021 was only 51.6% [141]. These poor outcomes highlight the need for new therapeutic strategies in light of ovarian cancer’s high heterogeneity and complex cellular origins [142].

Using the AutoDock Vina system, Abd Ghani et al. [143] examined the molecular interaction between flavonoids and anti-apoptotic proteins Bcl-2 and Bcl-xl [143]. Fisetin demonstrated the strongest binding affinity to Bcl-xl (−8.8 kcal/mol) via hydrophobic and electrostatic interactions, and a reasonable affinity to Bcl-2 (−7.1 kcal/mol) through electrostatic interactions with PHE63. These findings suggested that fisetin, like other flavonoids, may function as a pro-apoptotic agent by inhibiting anti-apoptotic proteins in ovarian cancer cells. Liu et al. [30] further investigated this possibility using ovarian cancer cell lines A2780 and OVCAR-3. Fisetin treatment significantly decreased cell viability in a dose-dependent manner at 25, 50, and 100 μM, as measured by MTT assay [30]. Annexin V/propidium iodide staining confirmed an increase in apoptosis, and real-time PCR demonstrated reduced mitochondrial cytochrome C mRNA levels [30], suggesting activation of the intrinsic apoptotic pathway. To assess whether alternative cell death pathways were involved, they used z-VAD, a pan-caspase inhibitor. Although z-VAD partially reduced fisetin-induced apoptosis, it did not restore cell proliferation to levels seen with z-VAD treatment alone [30], suggesting that additional mechanisms were contributing to cell death. Western blotting revealed increased expression of ZBP1, RIP3, and MLKL in fisetin-treated cells [30], indicating activation of necroptosis. Necroptosis is a regulated form of cell death implicated in immune clearance and tumor suppression. Dysregulation of necroptosis proteins, particularly MLKL, is associated with cancer progression and poorer survival in ovarian cancer [144,145].

Several studies have also explored its potential as a synergistic agent with platinum-based chemotherapy, which is a mainstay in ovarian cancer treatment along with surgical debulking [10]. Platinum resistance develops in many patients, especially those with recurrent disease, despite initial responsiveness in approximately 75% of individuals with high-grade serous ovarian carcinoma [146]. Koren Carmi et al. [124] demonstrated that co-culturing A2780 cells with murine or human mesenchymal stem cells induced resistance to the platinum prodrug RJY13. Fisetin (10 μM) restored platinum sensitivity by modulating ERK1/2 signaling. While mesenchymal stem cell co-culture reduced phospho-ERK1/2, fisetin treatment upregulated ERK phosphorylation, which reversed drug resistance [124]. Jafarzadeh et al. [125] showed that combined use of cisplatin (0.1 μg/mL or 0.5 μg/mL) and fisetin (50 μg/mL or 75 μg/mL or 100 μg/mL) significantly reduced the proportion of viable cells at all possible dose combinations, including when cisplatin was used at a lower concentration than its IC50 of 0.75 μg/mL.

Several studies have also explored drug delivery platforms to improve its therapeutic potential. For example, Xiao et al. [126] used polymeric micelles to encapsulate fisetin and demonstrated enhanced cytotoxicity and apoptosis induction in SKOV3 ovarian cancer cells compared with free fisetin. These results were confirmed in vivo using SKOV3 xenografts in BALB/c athymic nude mice, where encapsulated fisetin produced greater tumor suppression as measured by ultrasound imaging and TUNEL staining [126].

**Figure 6:**
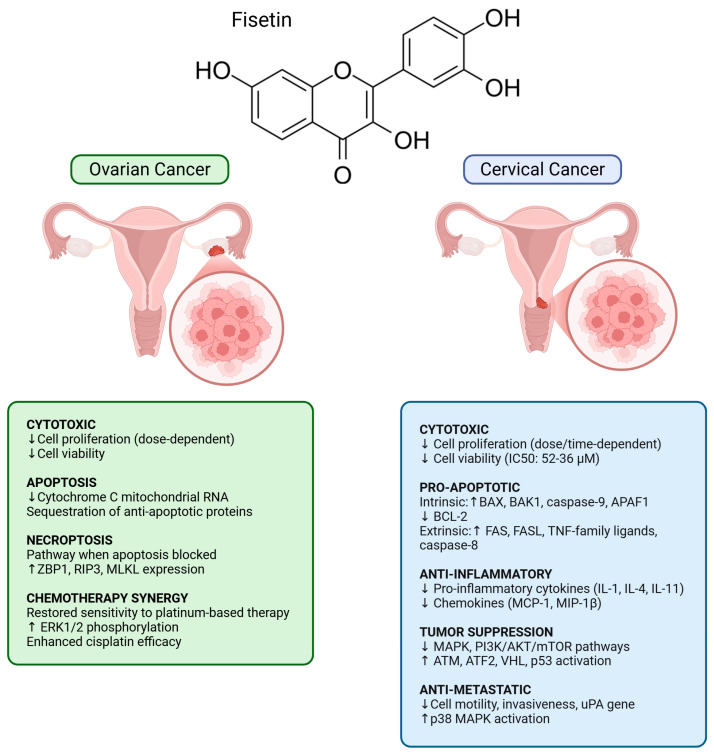
Anticancer effects of fisetin in ovarian and cervical cancers. Figures made in BioRender.com.

Overall, these studies suggest that fisetin exerts antitumor effects in ovarian cancer, including induction of apoptosis and necroptosis, inhibition of anti-apoptotic proteins, and reversal of chemotherapy resistance (Figure 6 and Table 1). Drug delivery systems further enhance fisetin’s therapeutic potential in ovarian cancer.

### 6.2. Cervical Cancer

Cervical cancer arises from malignant transformation of cells in the cervix, the lower and narrow portion of the uterus. Persistent infection with high-risk human papillomavirus (HPV) genotypes, particularly HPV-16 and HPV-18, accounts for the vast majority of cervical cancer cases globally [147]. Additional risk factors include smoking, a higher number of sexual partners, and immunosuppressive therapy [148]. In the United States, significant declines in cervical cancer incidence and mortality over the past two decades reflect successful public health measures such as cervical cancer screening and HPV vaccination programs [149]. Currently, treatment options include a combination of surgical resection, chemotherapy, and immunotherapy [150]. The overall five-year relative survival rate is 67% and may reach 91% for early-stage disease [151].

Fisetin has demonstrated potential anticancer activity in cervical cancer models (Figure 6 and Table 1). Afroze et al. [21] evaluated fisetin in HeLa cells and observed dose- and time-dependent inhibition of cellular proliferation compared with DMSO-treated controls. Fisetin induced apoptosis through both intrinsic and extrinsic pathways, as evidenced by increased expression of BAX, BAK1, caspase-9, and APAF1, along with decreased BCL-2 in the intrinsic pathway, and upregulation of FAS, FASL, TNF-family ligands, and caspase-8 in the extrinsic pathway [21]. Fisetin also exerted notable anti-inflammatory effects, reducing expression of cytokines (IL-1 family, IL-4, and IL-11) and chemokines (MCP-1 and MIP-1β) [21]. At a molecular level, fisetin downregulated key proliferative signaling pathways, including MAPK and PI3K/AKT/mTOR, and activated tumor-suppressive mechanisms through upregulation of ATM, ATF2, VHL, and p53 [21]. The earlier work by Ying et al. [127] similarly demonstrated fisetin’s ability to reduce HeLa cell viability in a time- and concentration-dependent manner, with IC50 values of 52 ± 0.9 μM at 24 h and 36 ± 0.5 μM at 48 h. Mechanistically, fisetin induced sustained ERK1/2 phosphorylation, which was associated with fisetin-mediated apoptosis [127]. In vivo validation using a nude mouse xenograft model revealed significantly reduced tumor growth in mice treated with fisetin compared to controls [127].

Beyond effects on proliferation and apoptosis, fisetin also exhibits anti-metastatic properties. Chou et al. [128] found that fisetin (10–40 μM) significantly inhibited motility and invasiveness of SiHa cervical cancer cells, with maximal effects at 20 and 40 μM. Fisetin also suppressed metastasis by inactivating p38 MAPK, blocking NF-κB nuclear translocation, and reducing expression of downstream targets such as urokinase-type plasminogen activator (uPA) [128]. uPA is known to promote cervical cancer invasion [152] and may serve as a biomarker for metastatic risk [153].

Similar to findings in ovarian cancer models, fisetin can act synergistically with targeted therapies. Lin et al. [129] demonstrated that combining fisetin (40 μM) with sorafenib (2.5 or 5 μM) significantly reduced HeLa cell viability compared with either agent alone. In vivo, HeLa xenografts in nude mice treated with fisetin (4 mg/kg), sorafenib (10 mg/kg), or their combination revealed that dual therapy produced the greatest reduction in tumor volume [129]. Mechanistically, the combination therapy upregulated death receptor 5 (DR5), enhanced activation of caspase-8 and caspase-3, and increased the Bax/Bcl-2 ratio [129].

Overall, these findings support the potential anticancer activity of fisetin in cervical cancer, including reduction of proliferation, induction of apoptosis, suppression of inflammation, inhibition of metastasis, and enhanced sensitivity to targeted therapies (Figure 6 and Table 1).

Importantly, these findings do not support off-label clinical use of fisetin in gynecologic oncology. No clinical trials have been conducted in these conditions, and human studies are needed to establish safety, optimal dosing, efficacy, and drug interactions.

## 7. Clinical Trials of Fisetin

Clinical trials evaluating fisetin in reproductive health and related diseases remain limited, with only two phase 2 studies (NCT06113016 and NCT05595499) conducted to date in breast cancer survivors (Table 2). The first, a randomized, placebo-controlled interventional trial (NCT06113016), is designed for women who have completed treatment for early-stage (stage I–III) breast cancer and aims to enroll 164 participants. In this study, participants receive fisetin as a nutritional supplement with a structured exercise and supportive-care/quality-of-life program, compared to a control arm receiving placebo plus the same exercise/support program. The trial began on 23 July 2024, with an estimated primary completion date of 30 June 2028 and study completion on 30 December 2028. The main objective is to assess whether the combination of fisetin and exercise can prevent or reduce frailty and functional decline in breast cancer survivors, potentially by eliminating senescent cells and reducing inflammation; secondary aims include improving physical performance, quality of life, and biomarkers of senescence. This trial is now recruiting. A second phase 2, randomized, double-blind, placebo-controlled trial (NCT05595499) is evaluating whether oral fisetin can improve physical function in post-chemotherapy survivors of stage I–III breast cancer. The study consists of two arms—fisetin versus placebo—with no additional drugs administered in combination. Although the ClinicalTrials.gov entry does not report the specific fisetin dose or duration of dosing, the overall study duration spans from baseline assessment to post-treatment functional evaluations. The trial began on 27 March 2023, with an estimated primary and final completion date of 1 June 2026, and aims to enroll 88 participants. The primary objective is to determine whether fisetin improves 6 min walk distance (6MWD) in frail breast cancer survivors, with secondary outcomes including grip strength, Short Physical Performance Battery (SPPB), frailty phenotype, quality-of-life measures, and additional functional or patient-reported outcomes. This trial is also recruiting, and no results have yet been reported. Although clinical trials of fisetin in reproductive health and related diseases are still limited, a significant number of trials have been completed or are ongoing in other health conditions which collectively support its potential efficacy and safety (Table 2). These include mild cognitive impairment (NCT02741804), Gulf War illness (NCT02909686), diabetic and chronic kidney disease (NCT03325322), frail elderly syndrome (NCT03675724), frailty and childhood cancer (NCT04733534), skeletal health (NCT04313634), COVID-19 (NCT04476953, NCT04771611, and NCT04537299), knee osteoarthritis (NCT04210986, NCT04815902, NCT05276895, NCT04770064, and NCT05482672), meniscus tear (NCT05505747), femoroacetabular impingement (NCT05025956), primary open-angle glaucoma (NCT04784234), carpal tunnel syndrome (NCT05416515), multimorbidity (NCT06431932), sepsis (NCT05758246), endothelial dysfunction and arterial stiffness (NCT06133634), and peripheral arterial disease (NCT06399809).

## 8. Limitations

A key limitation is that most in vitro concentrations exceed achievable plasma levels in humans. Across the studies reviewed, in vitro fisetin concentrations ranged from 5 to 300 μM (Table 1), with particularly high doses (up to 300 μM) frequently employed in gynecological cancer models [125,126]. In contrast, fisetin exhibits low oral bioavailability (approximately 7.8–31.7%) due to extensive phase II conjugation and rapid systemic metabolism [34,36]. Pharmacokinetic studies in rodents demonstrate that peak plasma concentrations of fisetin and its major metabolite, geraldol, remain in the low to mid-micromolar range even at high oral doses (up to 200 mg/kg) [36], indicating a critical pharmacokinetic gap between experimental and physiologically achievable concentrations.

In addition, although multiple clinical trials evaluating fisetin are registered or ongoing (Table 2), none is focused on gynecological conditions. As a result, the optimal dosing, long-term safety, and therapeutic efficacy of fisetin in reproductive tissues remain insufficient and require validation in well-designed clinical studies.

## 9. Conclusions and Future Perspectives

Across preclinical models, fisetin emerges as a multifunctional flavonoid with relevance to multiple conditions affecting women’s reproductive health. In ovarian aging and fertility, fisetin attenuates oxidative stress; improves mitochondrial function; modulates Sirt1 and Nrf2/HO-1 signaling; and reduces senescence markers in oocytes, granulosa cells, and ovarian tissue, suggesting potential to preserve gamete quality and delay functional decline. In menopause, its senolytic and antioxidant actions, together with preliminary evidence for bone-protective effects, highlight a possible role in improving skeletal and systemic consequences of estrogen loss. In benign gynecologic disorders such as endometriosis, uterine fibroids, and PCOS, fisetin consistently reduces inflammation, fibrosis, oxidative stress, and senescence, while improving hormonal, metabolic, and histological parameters in animal and cell culture models. In gynecologic malignancies, fisetin induces apoptosis and necroptosis, downregulates survival and proliferative pathways, inhibits migration and invasion, and enhances sensitivity to platinum agents and targeted therapies, particularly in ovarian and cervical cancer models.

However, important limitations temper these promising findings. Currently, existing evidence is largely based on in vitro or short-term animal studies. Clinical data are scarce and largely indirect, with no completed randomized trials specifically targeting reproductive or gynecologic indications, except two trials in breast cancer survivors. Pharmacokinetic challenges, including poor solubility, limited bioavailability, and uncertain tissue distribution, remain incompletely addressed, although nanoformulations and micellar delivery systems offer potential solutions. Many studies focus on short-term endpoints, long-term safety, or interactions with standard hormonal therapies, chemotherapeutics, or anticoagulants. In PCOS and benign disease models, comparisons with standard-of-care agents are still sparse.

Future research, including rigorous, well-designed trials, is needed. Key priorities include the following: (i) pharmacokinetic and pharmacodynamic studies in humans to define safe and effective dosing strategies, including advanced formulations to overcome hydrophobicity; (ii) early-phase clinical trials in well-defined populations, such as women with PCOS, endometriosis, fibroids, or perimenopausal symptoms, incorporating both symptom-based and biomarker outcomes (senescence, SASP, oxidative stress, and fibrosis); (iii) rigorous evaluation of fisetin as an adjunct to existing therapies, including chemotherapeutic and hormonal regimens, to clarify potential synergy or antagonism. Addressing these gaps will be essential to determine whether fisetin can transition from a promising experimental compound to a clinically useful, non-hormonal adjunct in women’s reproductive and gynecologic health.

## Figures and Tables

**Table 2:** Clinical trials of fisetin in different human conditions.

Category	Conditions	NCT Number	Phase	Sex	Age	Enrolled (n)	Dosing	Objectives	Location	Current Status
Aging and Frailty	Frail elderly syndrome	NCT03675724	Phase 2	All	≥70	40	Fisetin 20 mg/kg/day orally for 2 days	To evaluate whether fisetin reduces frailty, inflammation, insulin resistance, and bone resorption markers in older adults	Mayo Clinic in Rochester, Rochester, Minnesota, United States	Enrolling by invitation (2018–2027)
	Frail elderly syndrome	NCT03430037	Phase 2	Female	≥70	40	Fisetin 20 mg/kg/day orally for 2 days each month for 2 months	To assess whether fisetin reduces inflammation, insulin resistance, bone resorption, and frailty in older women with gait disturbance	Mayo Clinic in Rochester, Rochester, Minnesota, United States	Enrolling by invitation (2018–2027)
	Frailty, childhood cancer	NCT04733534	Phase 2	All	≥18	110	Arm 1 (Dasatinib + Quercetin): 100 mg dasatinib daily plus 500 mg quercetin twice daily on days 1–3 and 30–32. Arm 2 (Fisetin): 20 mg/kg/day on days 1–2 and 30–31	To evaluate whether short senolytic regimens (dasatinib + quercetin or fisetin) reduce cellular senescence and improve frailty in adult survivors of childhood cancer	St. Jude Children’s Research Hospital, Memphis, Tennessee, United States	Active, not recruiting (2022–2027)
	Skeletal health	NCT04313634	Phase 2	Female	≥60	74	Arm 1: Dasatinib + Quercetin: 100 mg dasatinib for 2 days plus 1000 mg quercetin daily for 3 days, repeated every 28 days for five cycles. Arm 2: Fisetin: ~20 mg/kg/day for 3 days, repeated every 28 days for five cycles	To assess whether senolytic treatment reduces senescent cell burden and favorably alters bone turnover markers in older women	Mayo Clinic in Rochester, Rochester, Minnesota, United States	Completed (2020–2023)
	Healthy aging	NCT07195318	Not applicable	All	≥50	120	Fisetin 100 mg orally once daily for 7 weeks	To evaluate the safety and potential anti-inflammatory and healthy-aging effects of daily low-dose (100 mg) fisetin supplementation over 7 weeks in middle-aged and older adults	Department of Clinical Research, Copenhagen University Hospital Amager & Hvidovre, Hvidovre, Denmark	Recruiting (2025–2035)
	Aging, endothelial dysfunction, arterial stiffness	NCT06133634	Phase 1 Phase 2	All	≥65	70	Fisetin 2 mg/kg/day for 3 days, repeated once after a 2-week interval	To determine whether intermittent fisetin treatment improves vascular endothelial function and reduces aortic stiffness in older adults while assessing underlying senescence-related mechanisms, safety, and tolerability	University of Colorado Boulder, Boulder, Colorado, United States	Active, not recruiting (2023–2027)
Musculoskeletal	Knee osteoarthritis	NCT04210986	Phase 1 Phase 2	All	40–80	75	20 mg/kg for 2 days, 28 days off, then another 2 days	To evaluate the safety of fisetin and determine whether it reduces senescent cells, inflammatory SASP markers, and osteoarthritis symptoms to improve joint function	The Steadman Clinic, Vail, Colorado, United States	Completed (2020–2023)
	Knee osteoarthritis	NCT04815902	Phase 1 Phase 2	All	40–85	100	Losartan 12.5 mg twice daily for 30 days starting the day after BMAC; fisetin 20 mg/kg on four pre-injection days and six post-injection days in three cycles	To evaluate whether fisetin and losartan, alone or combined, enhance the therapeutic effect of BMAC injections for knee osteoarthritis	The Steadman Clinic, Vail, Colorado, United States	Active, not recruiting (2021–2025)
	Knee osteoarthritis	NCT05276895	Not applicable	All	40–80	60	Arm 1: Quercetin + Fisetin: 1250 mg quercetin + 1000 mg fisetin daily for 3 days every 3 weeks over 12 weeks. Arm 2: Quercetin + Fisetin + Glycyrrhizin: 1250 mg quercetin + 1000 mg fisetin for 3 days, followed by 100 mg/day glycyrrhizin for 1 week every 3 weeks over 12 weeks	To evaluate whether natural senolytic agents alone or combined with NLRP3 inflammasome inhibition reduce knee pain and effusion-synovitis in symptomatic knee osteoarthritis	Assiut University, Faculty of Medicine, Asyut, Egypt	Suspended (2022–2024)
	Knee osteoarthritis	NCT04770064	Phase 1 Phase 2	All	35–80	60	High dose: 20 mg/kg for 2 days, a 28-day wash-out, then 2 more days; low dose: 100 mg daily for 90 days	To evaluate whether two fisetin dosing regimens reduce pain, improve function, and decrease senescence-related cartilage degradation in mild to moderate knee osteoarthritis	UK Healthcare at Turfland and UK HealthCare Joint Reconstruction and Replacement, Lexington, Kentucky, United States	Withdrawn (2023–2024)
	Knee osteoarthritis, obesity, depression	NCT05482672	Phase 2 Phase 3	All	≥40	120	Oral fisetin is taken for 2 days, followed by a 28-day washout, then another 2-day course	To test if the GetHealthy-OA program with fisetin improves pain and function in knee osteoarthritis with obesity and depression	UK Healthcare at Turfland and UK HealthCare Joint Reconstruction and Replacement, Lexington, Kentucky, United States	Withdrawn (2023–2023)
	Meniscus tear	NCT05505747	Phase 2 Phase 3	All	18–45	NA	20 mg/kg/day for 2 days, a 28-day washout, then another 2-day course, starting 8 weeks post-surgery	To test whether fisetin plus real-time biofeedback improves recovery and joint function after meniscus repair	UK Healthcare at Turfland, Lexington, Kentucky, United States	Withdrawn (2025–2026)
	Femoroacetabular impingement	NCT05025956	Phase 1 Phase 2	All	18–80	68	Fisetin 20 mg/kg/day for 2 days pre-surgery and on days 33–34, 63–64, and 93–94 post-surgery (100 mg capsules)	To assess whether perioperative fisetin improves the therapeutic effects of PRP and losartan in patients undergoing hip arthroscopy for femoroacetabular impingement or labral tear	The Steadman Clinic, Vail, Colorado, United States	Active, not recruiting (2021–2024)
	Carpal tunnel syndrome	NCT05416515	Phase 2	All	21–80	40	100 mg orally for two consecutive days, repeated once after one month	To evaluate the safety and effectiveness of fisetin for mild to moderate carpal tunnel syndrome	Mayo Clinic in Rochester, Rochester, Minnesota, United States	Active, not recruiting (2022–2025)
COVID-19	COVID-19	NCT04476953	Phase 2	All	≥18	80	Approximately 20 mg/kg/day given orally or via NG/D tube for 2 consecutive days	To evaluate whether fisetin can prevent worsening oxygenation, inflammation, and disease progression in hospitalized adults with COVID-19, while assessing its safety and tolerability	Mayo Clinic in Rochester, Rochester, Minnesota, United States	Active, not recruiting (2020–2026)
	COVID-19	NCT04771611	Phase 2	All	≥18	55	Approximately 20 mg/kg/day orally for four days total, given on days 0–1 and again on days 8–9	To evaluate whether short-term fisetin treatment reduces COVID-19–related complications and mortality while establishing its safety in at-risk outpatients	Mayo Clinic in Rochester, Rochester, Minnesota, United States	Completed (2021–2022)
	COVID-19	NCT04537299	Phase 2	All	≥65	20	Approximately 20 mg/kg/day given orally or via NG/D tube for 2 days, repeated on days 8 and 9	To evaluate whether fisetin can safely reduce disease progression and inflammation in older nursing-home residents with confirmed SARS-CoV-2 infection	Mayo Clinic in Rochester, Rochester, Minnesota, United States	Terminated (2022–2024)
Cancer and Survivorship	Breast cancer survivors	NCT06113016	Phase 2	Female	Child, adult, older adult	164	Fisetin taken orally on days 1–3 of each 14-day cycle for 8 cycles, plus a physical activity handout and blood sample collection	To evaluate whether fisetin, alone or combined with structured exercise, improves physical function and reduces frailty in postmenopausal breast cancer survivors after chemotherapy	UCLA Health Cancer Care in Alhambra, Alhambra; UCLA Health Beverly Hills Primary & Specialty Care, Beverly Hills; UCLA Health Burbank Primary & Specialty Care, Burbank; UCLA/Jonsson Comprehensive Cancer Center, Los Angeles; UCLA Health Primary Care in Marina del Rey, Marina del Rey; UCLA Health Primary Care in Pasadena, Pasadena, California, United States	Recruiting (2024–2028)
	Breast cancer survivors	NCT05595499	Phase 2	Female	Child, adult, older adult	88	Participants take oral fisetin on days 1–3 every 2 weeks for up to 8 weeks, with blood samples collected throughout the trial	To evaluate whether fisetin improves physical function—primarily the 6MWD in frail postmenopausal breast cancer survivors following chemotherapy	UCLA Health Cancer Care in Alhambra, Alhambra; UCLA Health Beverly Hills Primary & Specialty Care, Beverly Hills; UCLA Health Burbank Primary & Specialty Care, Burbank; City of Hope Comprehensive Cancer Center, Duarte; UCLA/Jonsson Comprehensive Cancer Center, Los Angeles; UCLA Health Primary Care in Marina del Rey, Marina del Rey; UCLA Health Primary Care in Pasadena, Pasadena, California, United States	Recruiting (2023–2026)
	Glioma	NCT07025226	Early phase 1	All	≥18	10	Not disclosed	To evaluate the safety, tolerability, and preliminary therapeutic activity of combining dasatinib, quercetin, fisetin, and temozolomide in patients with previously treated glioma with residual disease	Mayo Clinic in Rochester, Rochester, Minnesota, United States	Recruiting (2025–2027)
Chronic Diseases	Chronic kidney disease, diabetes, diabetic nephropathy	NCT03325322	Phase 2	All	40–80	26	Fisetin 20 mg/kg/day taken orally for 2 consecutive days	To evaluate whether a single 2-day course of oral fisetin improves stem cell function, kidney function, inflammation, and physical performance in individuals with advanced chronic kidney disease	Mayo Clinic in Rochester, Rochester, Minnesota, United States	Suspended (2018–2026)
	Peripheral arterial disease	NCT06399809	Phase 2	All	≥50	34	Fisetin at 20 mg/kg once daily for 2 days every 14 days, rounded to the nearest 100 mg capsule	To evaluate whether fisetin reduces senescent cell burden and improves 6MWD in older adults with peripheral artery disease, while exploring its effects on inflammatory and senescence biomarkers	Northwestern University Feinberg School of Medicine, Chicago, Illinois, United States	Recruiting (2024–2027)
	Common variable immunodeficiency, interstitial lung disease	NCT05593588	Phase 2	All	≥18	20	Fisetin is given at 20 mg/kg, supplied in 100 mg capsules, taken orally on days 0, 1, 28, and 29	To evaluate whether fisetin improves interstitial lung disease in individuals with common variable immunodeficiency compared with placebo	Mayo Clinic in Rochester, Rochester, Minnesota, United States	Enrolling by invitation (2023–2026)
Acute Conditions	Sepsis	NCT05758246	Phase 2	All	≥65	220	20 mg/kg once daily for 2 days	To identify the most effective dose of fisetin for reducing early organ failure in older patients with sepsis and to assess its potential for advancing to a definitive phase 3 trial	University of Florida, Gainesville, Florida; University of Iowa, Iowa City, Iowa; Ridges, Burnsville, Minnesota; Southdale, Edina, Minnesota; M Health Fairview St. John’s, Maplewood, Minnesota; St. John’s, Maplewood, Minnesota; University of Minnesota, Minneapolis, Minnesota; HCMC, Minneapolis, Minnesota; UMMC, Minneapolis, Minnesota; University of Mississippi Medical Center, Jackson, Mississippi, United States	Recruiting (2023–2026)
Neurological Conditions	Mild cognitive impairment	NCT02741804	Not applicable	All	≥55	150	BBH-1001 supplement (turmeric 125 mg, fisetin 16.65 mg, green tea extract 17.5 mg, EPA 75 mg, DHA 150 mg, vitamin D3 250 IU) as 4 daily softgels for 18 months	To evaluate whether a micronutrient supplement combined with a multi-domain lifestyle intervention can reduce retinal amyloid and improve cognitive outcomes in individuals with mild cognitive impairment	Cedars-Sinai Medical Center, Los Angeles, California, United States	Unknown (2016–2019)
	Gulf War illness	NCT02909686	Not applicable	Male	39–65	36	Fisetin dose: 200–800 mg orally once daily	To evaluate whether nine anti-inflammatory botanical compounds can reduce symptoms in Gulf War Illness	University of Alabama at Birmingham, Birmingham, Alabama, United States	Completed (2016–2022)
Others	Fatigue	NCT06819254	Phase 4	All	≥65	60	Participants first receive fisetin 20 mg/kg twice daily for 2 consecutive days each week over 2 weeks, followed by a 14-day washout, then cross-over to an identical 2-week placebo regimen	To evaluate whether short-course fisetin supplementation reduces fatigue in older adult cancer survivors compared with placebo in a randomized, double-blind, cross-over design	Atrium Health Wake Forest Baptist Hospital, Winston-Salem, North Carolina, United States	Not yet recruiting (2025–2026)
	Primary open-angle glaucoma	NCT04784234	Not applicable	All	40–80	100	Not disclosed	To determine whether six months of daily GlaucoCetin improves vision and visual function in patients with open-angle glaucoma compared with placebo	Wills Eye Hospital, Glaucoma Research Center, Philadelphia, Pennsylvania, United States	Unknown (2021–2023)
	Sleep disorder, aging	NCT06990256	Not applicable	All	45–70	80	Fisetin Group: participants take 500 mg of fisetin once daily after breakfast for 12 weeks. Combined Urolithin A + Fisetin Group: participants take 300 mg urolithin A + 200 mg fisetin once daily after breakfast for 12 weeks	To determine whether urolithin A, fisetin, or their combination improves sleep quality and aging-related biomarkers in middle-aged and older adults over a 12-week intervention	Wuchang Hospital Affiliated to Wuhan University of Science and Technology, Wuhan, Hubei, China	Not yet recruiting (2025–2026)
	Pharmacokinetic study in healthy volunteers	NCT06796374	Not applicable	All	≥18	80	FISEKIN-1 is a four-arm study comparing fisetin pharmacokinetics in young (18–30 years) and older adults (≥65 years) receiving either a single 100 mg fisetin capsule or a higher-dose formulation of 1000 mg fisetin plus 200 mg quercetin in two softgel capsules	To compare fisetin pharmacokinetics between young and older adults using two different oral fisetin formulations and doses	University Medicine Greifswald, Germany	Not yet recruiting (2025–2026)
	Multimorbidity	NCT06431932	Phase 1 Phase 2	All	≥20	60	Fisetin at 20 mg/kg/day for two consecutive days	To evaluate the pharmacokinetics, safety, and tolerability of fisetin and identify feasible biomarkers and outcome measures for future clinical trials in healthy adults and older patients	Copenhagen University Hospital, Amager and Hvidovre, Denmark	Not yet recruiting (2025–2034)

## Data Availability

No new data were created or analyzed in this study.

## Abbreviations

The following abbreviations are used in this manuscript:

AUC	Area Under the Curve
CAT	Catalase
FOXO3	Forkhead Box O3
GSH	Glutathione
GPX	Glutathione Peroxidase
HPV	Human Papillomavirus
LC3-II	Light Chain 3-II
LPS	Lipopolysaccharide
NOX1	NADPH Oxidase 1
PCOS	Polycystic Ovarian Syndrome
ROS	Reactive Oxygen Species
SAR	Structure–Activity Relationship
SASP	Senescence-Associated Secretory Phenotype
SNEDDSs	Self-Nanoemulsifying Systems
SPPB	Short Physical Performance Battery
SOD	Superoxide Dismutase
uPA	Urokinase-type Plasminogen Activator
6MWD	6 min Walk Distance

## References

[1] Falcone T., Flyckt R. (2018). Clinical management of endometriosis. Obstet. Gynecol..

[2] Stewart E.A., Cookson C., Gandolfo R.A., Schulze-Rath R. (2017). Epidemiology of uterine fibroids: A systematic review. BJOG Int. J. Obstet. Gynaecol..

[3] Palomba S., Santagni S., Falbo A., La Sala G.B. (2015). Complications and challenges associated with polycystic ovary syndrome: Current perspectives. Int. J. Women’s Health.

[4] Siegel R.L., Miller K.D., Jemal A. (2018). Cancer statistics, 2018. CA Cancer J. Clin..

[5] Islam M.S., Afrin S., Jones S.I., Segars J. (2020). Selective progesterone receptor modulators—Mechanisms and therapeutic utility. Endocr. Rev..

[6] Wekker V., Van Dammen L., Koning A., Heida K.Y., Painter R.C., Limpens J., Laven J.S.E., Roeters van Lennep J.E., Roseboom T.J., Hoek A. (2020). Long-term cardiometabolic disease risk in women with PCOS: A systematic review and meta-analysis. Hum. Reprod. Update.

[7] Yin X., Ji Y., Chan C.L.W., Chan C.H.Y. (2021). The mental health of women with polycystic ovary syndrome: A systematic review and meta-analysis. Arch. Women’s Ment. Health.

[8] Go V.A.A., Thomas M.C., Singh B., Prenatt S., Sims H., Blanck J.F., Segars J.H. (2020). A systematic review of the psychosocial impact of fibroids before and after treatment. Am. J. Obstet. Gynecol..

[9] Al-Hendy A., Myers E.R., Stewart E. (2017). Uterine fibroids: Burden and unmet medical need. Semin. Reprod. Med..

[10] Kuroki L., Guntupalli S.R. (2020). Treatment of epithelial ovarian cancer. BMJ.

[11] Christ J.P., Cedars M.I. (2023). Current guidelines for diagnosing PCOS. Diagnostics.

[12] Ramos-Nino M.E. (2025). Non-Hormonal strategies in endometriosis: Targets with future clinical potential. J. Clin. Med..

[13] Islam M.S., Parish M., Brennan J.T., Winer B.L., Segars J.H. (2023). Targeting fibrotic signaling pathways by EGCG as a therapeutic strategy for uterine fibroids. Sci. Rep..

[14] Parish M., Massoud G., Hazimeh D., Segars J., Islam M.S. (2023). Green Tea in Reproductive cancers: Could Treatment be as simple?. Cancers.

[15] Hazimeh D., Massoud G., Parish M., Singh B., Segars J., Islam M.S. (2023). Green tea and benign gynecologic disorders: A new trick for an old beverage?. Nutrients.

[16] Li W., Qin L., Feng R., Hu G., Sun H., He Y., Zhang R. (2019). Emerging senolytic agents derived from natural products. Mech. Ageing Dev..

[17] Arai Y., Watanabe S., Kimira M., Shimoi K., Mochizuki R., Kinae N. (2000). Dietary intakes of flavonols, flavones and isoflavones by Japanese women and the inverse correlation between quercetin intake and plasma LDL cholesterol concentration. J. Nutr..

[18] Park H.-H., Lee S., Oh J.-M., Lee M.-S., Yoon K.-H., Park B.H., Kim J.W., Song H., Kim S.-H. (2007). Anti-inflammatory activity of fisetin in human mast cells (HMC-1). Pharmacol. Res..

[19] Dong B., Liu C., Xue R., Wang Y., Sun Y., Liang Z., Fan W., Jiang J., Zhao J., Su Q. (2018). Fisetin inhibits cardiac hypertrophy by suppressing oxidative stress. J. Nutr. Biochem..

[20] Arangia A., Marino Y., Fusco R., Siracusa R., Cordaro M., D’Amico R., Macrì F., Raffone E., Impellizzeri D., Cuzzocrea S. (2023). Fisetin, a natural polyphenol, ameliorates endometriosis modulating mast cells derived NLRP-3 inflammasome pathway and oxidative stress. Int. J. Mol. Sci..

[21] Afroze N., Pramodh S., Shafarin J., Bajbouj K., Hamad M., Sundaram M.K., Haque S., Hussain A. (2022). Fisetin deters cell proliferation, induces apoptosis, alleviates oxidative stress and inflammation in human cancer cells, HeLa. Int. J. Mol. Sci..

[22] Zhu Y., Doornebal E.J., Pirtskhalava T., Giorgadze N., Wentworth M., Fuhrmann-Stroissnigg H., Niedernhofer L.J., Robbins P.D., Tchkonia T., Kirkland J.L. (2017). New agents that target senescent cells: The flavone, fisetin, and the BCL-XL inhibitors, A1331852 and A1155463. Aging.

[23] Tavenier J., Nehlin J.O., Houlind M.B., Rasmussen L.J., Tchkonia T., Kirkland J.L., Andersen O., Rasmussen L.J.H. (2024). Fisetin as a senotherapeutic agent: Evidence and perspectives for age-related diseases. Mech. Ageing Dev..

[24] Wu J., Liu Y., Song Y., Wang L., Ai J., Li K. (2022). Aging conundrum: A perspective for ovarian aging. Front. Endocrinol..

[25] Fu Y., Cao Y., Yan Y., Huang S., Li S., Huang Y., Wang Z., Gao L., Xiao C. (2024). An in-depth overview of the molecular mechanisms governing ovarian aging and the corresponding preventative and therapeutic strategies. Aging Res..

[26] Kirchner V.A., Badshah J.S., Hong S.K., Martinez O., Pruett T.L., Niedernhofer L.J. (2024). Effect of cellular senescence in disease progression and transplantation: Immune cells and solid organs. Transplantation.

[27] Islam M.S., Ciavattini A., Petraglia F., Castellucci M., Ciarmela P. (2018). Extracellular matrix in uterine leiomyoma pathogenesis: A potential target for future therapeutics. Hum. Reprod. Update.

[28] Mihanfar A., Nouri M., Roshangar L., Khadem-Ansari M.H. (2021). Ameliorative effects of fisetin in letrozole-induced rat model of polycystic ovary syndrome. J. Steroid Biochem. Mol. Biol..

[29] Lee J.-W., Choi H.J., Kim E.-J., Hwang W.Y., Jung M.-H., Kim K.S. (2020). Fisetin induces apoptosis in uterine leiomyomas through multiple pathways. Sci. Rep..

[30] Liu Y., Cao H., Zhao Y., Shan L., Lan S. (2022). Fisetin-induced cell death in human ovarian cancer cell lines via zbp1-mediated necroptosis. J. Ovarian Res..

[31] Herzig J. (1891). Studien über Quercetin und seine Derivate: VII. Abhandlung. Monatshefte Chem. Verwandte Teile Anderer Wiss..

[32] Kostanecki S.V., Lampe V., Tambor J. (1904). Synthese des fisetins. Berichte Dtsch. Chem. Ges..

[33] Lee S.-O., Kim S.-J., Kim J.-S., Ji H., Lee E.-O., Lee H.-J. (2018). Comparison of the main components and bioactivity of *Rhus verniciflua* Stokes extracts by different detoxification processing methods. BMC Complement. Altern. Med..

[34] Huang M.-C., Hsueh T.Y., Cheng Y.-Y., Lin L.-C., Tsai T.-H. (2018). Pharmacokinetics and biliary excretion of fisetin in rats. J. Agric. Food Chem..

[35] Zhang X., Yin J., Liang C., Sun Y., Zhang L. (2017). UHPLC-Q-TOF-MS/MS method based on four-step strategy for metabolism study of fisetin in vitro and in vivo. J. Agric. Food Chem..

[36] Jo J.H., Jo J.J., Lee J.-M., Lee S. (2016). Identification of absolute conversion to geraldol from fisetin and pharmacokinetics in mouse. J. Chromatogr. B.

[37] Touil Y.S., Auzeil N., Boulinguez F., Saighi H., Regazzetti A., Scherman D., Chabot G.G. (2011). Fisetin disposition and metabolism in mice: Identification of geraldol as an active metabolite. Biochem. Pharmacol..

[38] Seguin J., Brullé L., Boyer R., Lu Y.M., Romano M.R., Touil Y.S., Scherman D., Bessodes M., Mignet N., Chabot G.G. (2013). Liposomal encapsulation of the natural flavonoid fisetin improves bioavailability and antitumor efficacy. Int. J. Pharm..

[39] Ragelle H., Crauste-Manciet S., Seguin J., Brossard D., Scherman D., Arnaud P., Chabot G.G. (2012). Nanoemulsion formulation of fisetin improves bioavailability and antitumour activity in mice. Int. J. Pharm..

[40] Bothiraja C., Yojana B.D., Pawar A.P., Shaikh K.S., Thorat U.H. (2014). Fisetin-loaded nanocochleates: Formulation, characterisation, in vitro anticancer testing, bioavailability and biodistribution study. Expert Opin. Drug Deliv..

[41] Liu W.-Y., Lin C.-C., Hsieh Y.-S., Wu Y.-T. (2021). Nanoformulation development to improve the biopharmaceutical properties of fisetin using design of experiment approach. Molecules.

[42] Kumar R., Kumar R., Khurana N., Singh S.K., Khurana S., Verma S., Sharma N., Kapoor B., Vyas M., Khursheed R. (2020). Enhanced oral bioavailability and neuroprotective effect of fisetin through its SNEDDS against rotenone-induced Parkinson’s disease rat model. Food Chem. Toxicol..

[43] Krishnakumar I.M., Jaja-Chimedza A., Joseph A., Balakrishnan A., Maliakel B., Swick A. (2022). Enhanced bioavailability and pharmacokinetics of a novel hybrid-hydrogel formulation of fisetin orally administered in healthy individuals: A randomised double-blinded comparative crossover study. J. Nutr. Sci..

[44] Markovic Z.S., Mentus S.V., Dimitrić Marković J.M. (2009). Electrochemical and density functional theory study on the reactivity of fisetin and its radicals: Implications on in vitro antioxidant activity. J. Phys. Chem. A.

[45] Ishige K., Schubert D., Sagara Y. (2001). Flavonoids protect neuronal cells from oxidative stress by three distinct mechanisms. Free Radic. Biol. Med..

[46] Lee S.E., Jeong S.I., Yang H., Park C.S., Jin Y.H., Park Y.S. (2011). Fisetin induces Nrf2-mediated HO-1 expression through PKC-δ and p38 in human umbilical vein endothelial cells. J. Cell. Biochem..

[47] Zhang H., Zheng W., Feng X., Yang F., Qin H., Wu S., Hou D.-X., Chen J. (2019). Nrf2–ARE signaling acts as master pathway for the cellular antioxidant activity of fisetin. Molecules.

[48] Prasath G.S., Sundaram C.S., Subramanian S.P. (2013). Fisetin averts oxidative stress in pancreatic tissues of streptozotocin-induced diabetic rats. Endocrine.

[49] Zhang L., Wang H., Zhou Y., Zhu Y., Fei M. (2018). Fisetin alleviates oxidative stress after traumatic brain injury via the Nrf2-ARE pathway. Neurochem. Int..

[50] Piao M.J., Kim K.C., Chae S., Keum Y.S., Kim H.S., Hyun J.W. (2013). Protective effect of fisetin (3,7,3′,4′-tetrahydroxyflavone) against γ-irradiation-induced oxidative stress and cell damage. Biomol. Ther..

[51] Lian T.-W., Wang L., Lo Y.-H., Huang I.-J., Wu M.-J. (2008). Fisetin, morin and myricetin attenuate CD36 expression and oxLDL uptake in U937-derived macrophages. Biochim. Biophys. Acta (BBA)-Mol. Cell Biol. Lipids.

[52] Burda S., Oleszek W. (2001). Antioxidant and antiradical activities of flavonoids. J. Agric. Food Chem..

[53] Firuzi O., Lacanna A., Petrucci R., Marrosu G., Saso L. (2005). Evaluation of the antioxidant activity of flavonoids by “ferric reducing antioxidant power” assay and cyclic voltammetry. Biochim. Biophys. Acta (BBA)-Gen. Subj..

[54] Kim S.-C., Kang S.-H., Jeong S.-J., Kim S.-H., Ko H.S., Kim S.-H. (2012). Inhibition of c-Jun N-terminal kinase and nuclear factor κ B pathways mediates fisetin-exerted anti-inflammatory activity in lipopolysccharide-treated RAW264.7 cells. Immunopharmacol. Immunotoxicol..

[55] Kim J.H., Kim M.-Y., Kim J.-H., Cho J.Y. (2015). Fisetin suppresses macrophage-mediated inflammatory responses by blockade of Src and Syk. Biomol. Ther..

[56] Zheng L.T., Ock J., Kwon B.-M., Suk K. (2008). Suppressive effects of flavonoid fisetin on lipopolysaccharide-induced microglial activation and neurotoxicity. Int. Immunopharmacol..

[57] Sun Y., Qin H., Zhang H., Feng X., Yang L., Hou D.-X., Chen J. (2021). Fisetin inhibits inflammation and induces autophagy by mediating PI3K/AKT/mTOR signaling in LPS-induced RAW264.7 cells. Food Nutr. Res..

[58] Gutiérrez-Venegas G., Contreras-Sánchez A., Ventura-Arroyo J.A. (2014). Anti-inflammatory activity of fisetin in human gingival fibroblasts treated with lipopolysaccharide. J. Asian Nat. Prod. Res..

[59] Peng H.-L., Huang W.-C., Cheng S.-C., Liou C.-J. (2018). Fisetin inhibits the generation of inflammatory mediators in interleukin-1β–induced human lung epithelial cells by suppressing the NF-κB and ERK1/2 pathways. Int. Immunopharmacol..

[60] Wu S.-J., Huang W.-C., Cheng C.-Y., Wang M.-C., Cheng S.-C., Liou C.-J. (2022). Fisetin suppresses the inflammatory response and oxidative stress in bronchial epithelial cells. Nutrients.

[61] Kwak S., Ku S.-K., Bae J.-S. (2014). Fisetin inhibits high-glucose-induced vascular inflammation in vitro and in vivo. Inflamm. Res..

[62] Sahu B.D., Kumar J.M., Sistla R. (2016). Fisetin, a dietary flavonoid, ameliorates experimental colitis in mice: Relevance of NF-κB signaling. J. Nutr. Biochem..

[63] Huang W., Li M.-L., Xia M.-Y., Shao J.-Y. (2018). Fisetin-treatment alleviates airway inflammation through inhbition of MyD88/NF-κB signaling pathway. Int. J. Mol. Med..

[64] Goh F.Y., Upton N., Guan S., Cheng C., Shanmugam M.K., Sethi G., Leung B.P., Wong W.F. (2012). Fisetin, a bioactive flavonol, attenuates allergic airway inflammation through negative regulation of NF-κB. Eur. J. Pharmacol..

[65] Ren Q., Guo F., Tao S., Huang R., Ma L., Fu P. (2020). Flavonoid fisetin alleviates kidney inflammation and apoptosis via inhibiting Src-mediated NF-κB p65 and MAPK signaling pathways in septic AKI mice. Biomed. Pharmacother..

[66] Pu J.-L., Huang Z.-T., Luo Y.-H., Mou T., Li T.-T., Li Z.-T., Wei X.-F., Wu Z.-J. (2021). Fisetin mitigates hepatic ischemia-reperfusion injury by regulating GSK3β/AMPK/NLRP3 inflammasome pathway. Hepatobiliary Pancreat. Dis. Int..

[67] Kim H.J., Kim S.H., Yun J.-M. (2012). Fisetin inhibits hyperglycemia-induced proinflammatory cytokine production by epigenetic mechanisms. Evid.-Based Complement. Altern. Med..

[68] Kim A., Yun J.-M. (2017). Combination treatments with luteolin and fisetin enhance anti-inflammatory effects in high glucose-treated THP-1 cells through histone acetyltransferase/histone deacetylase regulation. J. Med. Food.

[69] Zheng W., Feng Z., You S., Zhang H., Tao Z., Wang Q., Chen H., Wu Y. (2017). Fisetin inhibits IL-1β-induced inflammatory response in human osteoarthritis chondrocytes through activating SIRT1 and attenuates the progression of osteoarthritis in mice. Int. Immunopharmacol..

[70] Nabizadeh Z., Nasrollahzadeh M., Shabani A.A., Mirmohammadkhani M., Nasrabadi D. (2023). Evaluation of the anti-inflammatory activity of fisetin-loaded nanoparticles in an in vitro model of osteoarthritis. Sci. Rep..

[71] Farsad-Naeimi A., Alizadeh M., Esfahani A., Aminabad E.D. (2018). Effect of fisetin supplementation on inflammatory factors and matrix metalloproteinase enzymes in colorectal cancer patients. Food Funct..

[72] da Silva D.C., Jervis P.J., Martins J.A., Valentão P., Ferreira P.M., Pereira D.M. (2023). Fisetin derivatives exhibit enhanced anti-inflammatory activity and modulation of endoplasmic reticulum stress. Int. Immunopharmacol..

[73] Di Micco R., Krizhanovsky V., Baker D., d’Adda di Fagagna F. (2021). Cellular senescence in ageing: From mechanisms to therapeutic opportunities. Nat. Rev. Mol. Cell Biol..

[74] Acosta J.C., Banito A., Wuestefeld T., Georgilis A., Janich P., Morton J.P., Athineos D., Kang T.-W., Lasitschka F., Andrulis M. (2013). A complex secretory program orchestrated by the inflammasome controls paracrine senescence. Nat. Cell Biol..

[75] Basisty N., Kale A., Jeon O.H., Kuehnemann C., Payne T., Rao C., Holtz A., Shah S., Sharma V., Ferrucci L. (2020). A proteomic atlas of senescence-associated secretomes for aging biomarker development. PLoS Biol..

[76] Saliev T., Singh P.B. (2025). Targeting Senescence: A Review of Senolytics and Senomorphics in Anti-Aging Interventions. Biomolecules.

[77] Yousefzadeh M.J., Zhu Y., McGowan S.J., Angelini L., Fuhrmann-Stroissnigg H., Xu M., Ling Y.Y., Melos K.I., Pirtskhalava T., Inman C.L. (2018). Fisetin is a senotherapeutic that extends health and lifespan. eBioMedicine.

[78] Ji X.M., Dong X.X., Li J.P., Tai G.J., Qiu S., Wei W., Silumbwe C.W., Damdinjav D., Otieno J.N., Li X.X. (2025). Fisetin Clears Senescent Cells Through the Pi3k-Akt-Bcl-2/Bcl-xl Pathway to Alleviate Diabetic Aortic Aging. Phytother. Res..

[79] Mahoney S.A., Venkatasubramanian R., Darrah M.A., Ludwig K.R., VanDongen N.S., Greenberg N.T., Longtine A.G., Hutton D.A., Brunt V.E., Campisi J. (2024). Intermittent supplementation with fisetin improves arterial function in old mice by decreasing cellular senescence. Aging Cell.

[80] Ijima S., Saito Y., Nagaoka K., Yamamoto S., Sato T., Miura N., Iwamoto T., Miyajima M., Chikenji T.S. (2022). Fisetin reduces the senescent tubular epithelial cell burden and also inhibits proliferative fibroblasts in murine lupus nephritis. Front. Immunol..

[81] Zhang L., Tong X., Huang J., Wu M., Zhang S., Wang D., Liu S., Fan H. (2020). Fisetin alleviated bleomycin-induced pulmonary fibrosis partly by rescuing alveolar epithelial cells from senescence. Front. Pharmacol..

[82] Huard C.A., Gao X., Dey Hazra M.E., Dey Hazra R.-O., Lebsock K., Easley J.T., Millett P.J., Huard J. (2023). Effects of Fisetin treatment on cellular senescence of various tissues and organs of old sheep. Antioxidants.

[83] Hambright W.S., Mu X., Gao X., Guo P., Kawakami Y., Mitchell J., Mullen M., Nelson A.-L., Bahney C., Nishimura H. (2023). The senolytic drug fisetin attenuates bone degeneration in the Zmpste24−/− progeria mouse model. J. Osteoporos..

[84] Zhao R., Kou H., Jiang D., Wang F. (2023). Exploring the anti-aging effects of fisetin in telomerase-deficient progeria mouse model. PeerJ.

[85] Russo M., Moccia S., Luongo D., Russo G.L. (2023). Senolytic flavonoids enhance type-I and type-II cell death in human radioresistant colon cancer cells through AMPK/MAPK pathway. Cancers.

[86] Colman R., Tchkonia T., Pirtskhalava T., Giorgadze N., Prata L., Schaefer K., Kirkland J. (2020). Effect of Combined Dasatinib and Fisetin Treatment on Senescent Cell Clearance in Monkeys. Innov. Aging.

[87] Ashiqueali S.A., Chaudhari D., Zhu X., Noureddine S., Siddiqi S., Garcia D.N., Gostynska A., Stawny M., Rubis B., Zanini B.M. (2024). Fisetin modulates the gut microbiota alongside biomarkers of senescence and inflammation in a DSS-induced murine model of colitis. GeroScience.

[88] Jacob J., Aggarwal A., Bhattacharyya S., Sahni D., Sharma V., Aggarwal A. (2025). Fisetin and resveratrol exhibit senotherapeutic effects and suppress cellular senescence in osteoarthritic cartilage-derived chondrogenic progenitor cells. Eur. J. Pharmacol..

[89] Henschke A., Grześkowiak B., Ivashchenko O., Sánchez-Cerviño M.C., Coy E., Moya S. (2025). Targeting Cellular Senescence with Liposome-Encapsulated Fisetin: Evidence of Senomorphic Effect. Int. J. Mol. Sci..

[90] Kim S.G., Sung J.Y., Kim J.-R., Choi H.C. (2021). Fisetin-induced PTEN expression reverses cellular senescence by inhibiting the mTORC2-Akt Ser473 phosphorylation pathway in vascular smooth muscle cells. Exp. Gerontol..

[91] Fang Y., Medina D., Stockwell R., McFadden S., Quinn K., Peck M.R., Bartke A., Hascup K.N., Hascup E.R. (2023). Sexual dimorphic metabolic and cognitive responses of C57BL/6 mice to Fisetin or Dasatinib and quercetin cocktail oral treatment. GeroScience.

[92] Park S.U., Walsh L., Berkowitz K.M. (2021). Mechanisms of ovarian aging. Reproduction.

[93] Schummers L., Hutcheon J.A., Hacker M.R., VanderWeele T.J., Williams P.L., McElrath T.F., Hernandez-Diaz S. (2018). Absolute risks of obstetric outcomes risks by maternal age at first birth: A population-based cohort. Epidemiology.

[94] Lim S.W., Jin L., Luo K., Jin J., Shin Y.J., Hong S.Y., Yang C.W. (2017). Klotho enhances FoxO3-mediated manganese superoxide dismutase expression by negatively regulating PI3K/AKT pathway during tacrolimus-induced oxidative stress. Cell Death Dis..

[95] Hirano M., Onodera T., Takasaki K., Takahashi Y., Ichinose T., Nishida H., Hiraike H., Nagasaka K. (2025). Ovarian aging: Pathophysiology and recent developments in maintaining ovarian reserve. Front. Endocrinol..

[96] Yang Z., Zhang J., Yuan Q., Wang X., Zeng W., Mi Y., Zhang C. (2024). Flavonoid Fisetin Alleviates Ovarian Aging of Laying Chickens by Enhancing Antioxidant Capacity and Glucose Metabolic Homeostasis. Antioxidants.

[97] Dong J., Yang Z., Yuan Q., Zeng W., Mi Y., Zhang C. (2025). Preventive Effect of Fisetin on Follicular Granulosa Cells Senescence via Attenuating Oxidative Stress and Upregulating the Wnt/β-Catenin Signaling Pathway. Cells.

[98] Xing X., Liang Y., Li Y., Zhao Y., Zhang Y., Li Z., Li Z., Wu Z. (2023). Fisetin delays postovulatory oocyte aging by regulating oxidative stress and mitochondrial function through Sirt1 pathway. Molecules.

[99] Garcia D.N., Hense J.D., Zanini B.M., Isola J.V.V., Prosczek J.B., Ashiqueali S., Oliveira T.L., Mason J.B., Schadock I.C., Barros C.C. (2024). Senolytic treatment fails to improve ovarian reserve or fertility in female mice. GeroScience.

[100] Penzias A., Azziz R., Bendikson K., Falcone T., Hansen K., Hill M., Jindal S., Kalra S., Mersereau J., Reindollar R. (2022). Optimizing natural fertility: A committee opinion. Fertil. Steril..

[101] Wilcox A.J., Dunson D., Baird D.D. (2000). The timing of the “fertile window” in the menstrual cycle: Day specific estimates from a prospective study. BMJ.

[102] Sharma R., Biedenharn K.R., Fedor J.M., Agarwal A. (2013). Lifestyle factors and reproductive health: Taking control of your fertility. Reprod. Biol. Endocrinol..

[103] Alam F., Syed H., Amjad S., Baig M., Khan T.A., Rehman R. (2021). Interplay between oxidative stress, SIRT1, reproductive and metabolic functions. Curr. Res. Physiol..

[104] Ezati D., Vardiyan R., Talebi A.R., Alipour F., Pahang H., Mohammadi S. (2025). Fisetin attenuates the adverse effects of freezing and thawing procedures on the biological characteristics of human asthenoteratozoospermia samples. Reprod. Biol..

[105] Talaulikar V. (2022). Menopause transition: Physiology and symptoms. Best Pract. Res. Clin. Obstet. Gynaecol..

[106] Santoro N., Epperson C.N., Mathews S.B. (2015). Menopausal symptoms and their management. Endocrinol. Metab. Clin..

[107] Figueras F., Castelo-Branco C., Pons F., Sanjuán A., Vanrell J. (2001). Effect of continuous and sequential oral estrogen–progestogen replacement regimens on postmenopausal bone loss: A 2-year prospective study. Eur. J. Obstet. Gynecol. Reprod. Biol..

[108] Vogelvang T.E., Mijatovic V., Kenemans P., Teerlink T., van der Mooren M.J. (2004). HMR 3339, a novel selective estrogen receptor modulator, reduces total cholesterol, low-density lipoprotein cholesterol, and homocysteine in healthy postmenopausal women. Fertil. Steril..

[109] Ghosh M., Rodriguez-Garcia M., Wira C.R. (2014). The immune system in menopause: Pros and cons of hormone therapy. J. Steroid Biochem. Mol. Biol..

[110] Stevenson J.C., Ren M., Kahler E., Custodio M.G., Nappi R.E., Tatarchuk T., Simoncini T., Karpova V., Yu Q. (2024). Ultra-low dose estradiol and dydrogesterone for the treatment of menopausal symptoms in a pooled, multi-ethnic population. Maturitas.

[111] Nageswari C., Meena N., Gupta S., Thillaieaswaran B. (2025). Effect of Pilates Exercises on Pain, Endurance, Quality-of-Life, and Disability in Postmenopausal Women with Low Back Pain. Musculoskelet. Care.

[112] Khan N., Syed D.N., Ahmad N., Mukhtar H. (2013). Fisetin: A dietary antioxidant for health promotion. Antioxid. Redox Signal..

[113] Pašalić E., Tambuwala M.M., Hromić-Jahjefendić A. (2023). Endometriosis: Classification, pathophysiology, and treatment options. Pathol.-Res. Pract..

[114] Fryer J., Mason-Jones A.J., Woodward A. (2025). Understanding diagnostic delay for endometriosis: A scoping review using the social-ecological framework. Health Care Women Int..

[115] Ozkan S., Murk W., Arici A. (2008). Endometriosis and infertility: Epidemiology and evidence-based treatments. Ann. N. Y. Acad. Sci..

[116] As-Sanie S., Mackenzie S.C., Morrison L., Schrepf A., Zondervan K.T., Horne A.W., Missmer S.A. (2025). Endometriosis: A review. JAMA.

[117] Szypłowska M., Tarkowski R., Kułak K. (2023). The impact of endometriosis on depressive and anxiety symptoms and quality of life: A systematic review. Front. Public Health.

[118] Ou Y., Wang H., Zhou C., Chen Y., Lyu J., Feng M., Huang X. (2025). Endometriosis-associated infertility: Multi-omics insights into pathogenesis and precision therapeutics. Front. Endocrinol..

[119] Salker M., Teklenburg G., Molokhia M., Lavery S., Trew G., Aojanepong T., Mardon H.J., Lokugamage A.U., Rai R., Landles C. (2010). Natural selection of human embryos: Impaired decidualization of endometrium disables embryo-maternal interactions and causes recurrent pregnancy loss. PLoS ONE.

[120] Shih A.J., Adelson R.P., Vashistha H., Khalili H., Nayyar A., Puran R., Herrera R., Chatterjee P.K., Lee A.T., Truskinovsky A.M. (2022). Single-cell analysis of menstrual endometrial tissues defines phenotypes associated with endometriosis. BMC Med..

[121] Delenko J., Hyman N., Chatterjee P.K., Safaric Tepes P., Shih A.J., Xue X., Gurney J., Baker A.G., Wei C., Munoz Espin D. (2025). Targeting Cellular Senescence to Enhance Human Endometrial Stromal Cell Decidualization and Inhibit Their Migration. Biomolecules.

[122] Moustafa P.E., Abo El Nasr N.M.E., Shabana M.E., Saleh D.O. (2024). Fisetin mitigates letrozole-induced polycystic ovarian syndrome in rats: Crosstalk of AMPK/PI3K/AKT-mediated-Nrf2 antioxidant defense mechanism and the inflammasome NLRP3/NF-κB P65/IL-1β signaling pathways. Naunyn-Schmiedeberg’s Arch. Pharmacol..

[123] Chahal S.K., Kabra A. (2024). Fisetin ameliorates polycystic ovary syndrome in rats via a mechanistic modulation of AMP-activated protein kinase and SIRT1 molecular pathway. Naunyn-Schmiedeberg’s Arch. Pharmacol..

[124] Koren Carmi Y., Mahmoud H., Khamaisi H., Adawi R., Gopas J., Mahajna J. (2020). Flavonoids restore platinum drug sensitivity to ovarian carcinoma cells in a phospho-ERK1/2-dependent fashion. Int. J. Mol. Sci..

[125] Jafarzadeh S., Baharara J., Tehranipour M. (2021). Apoptosis induction with combined use of cisplatin and fisetin in cisplatin-resistant ovarian cancer cells (A2780). Avicenna J. Med. Biotechnol..

[126] Xiao X., Zou J., Fang Y., Meng Y., Xiao C., Fu J., Liu S., Bai P., Yao Y. (2018). Fisetin and polymeric micelles encapsulating fisetin exhibit potent cytotoxic effects towards ovarian cancer cells. BMC Complement. Altern. Med..

[127] Ying T.-H., Yang S.-F., Tsai S.-J., Hsieh S.-C., Huang Y.-C., Bau D.-T., Hsieh Y.-H. (2012). Fisetin induces apoptosis in human cervical cancer HeLa cells through ERK1/2-mediated activation of caspase-8-/caspase-3-dependent pathway. Arch. Toxicol..

[128] Chou R.-H., Hsieh S.-C., Yu Y.-L., Huang M.-H., Huang Y.-C., Hsieh Y.-H. (2013). Fisetin inhibits migration and invasion of human cervical cancer cells by down-regulating urokinase plasminogen activator expression through suppressing the p38 MAPK-dependent NF-κB signaling pathway. PLoS ONE.

[129] Lin M.-T., Lin C.-L., Lin T.-Y., Cheng C.-W., Yang S.-F., Lin C.-L., Wu C.-C., Hsieh Y.-H., Tsai J.-P. (2016). Synergistic effect of fisetin combined with sorafenib in human cervical cancer HeLa cells through activation of death receptor-5 mediated caspase-8/caspase-3 and the mitochondria-dependent apoptotic pathway. Tumor Biol..

[130] Dolmans M.-M., Petraglia F., Catherino W.H., Donnez J. (2024). Pathogenesis of uterine fibroids: Current understanding and future directions. Fertil. Steril..

[131] Boos D., Chuang T.-D., Abbasi A., Luzzi A., Khorram O. (2024). The immune landscape of uterine fibroids as determined by mass cytometry. F&S Sci..

[132] Lakabi R., Harth S., Meinhold-Heerlein I., Olsthoorn A.V., Munro M.G., Murji A. (2025). Diagnosis and classification of uterine fibroids. Int. J. Gynecol. Obstet..

[133] Giuliani E., As-Sanie S., Marsh E.E. (2020). Epidemiology and management of uterine fibroids. Int. J. Gynecol. Obstet..

[134] The Rotterdam ESHRE/ASRM-Sponsored PCOS Consensus Workshop Group (2004). Revised 2003 consensus on diagnostic criteria and long-term health risks related to polycystic ovary syndrome. Fertil. Steril..

[135] Salari N., Nankali A., Ghanbari A., Jafarpour S., Ghasemi H., Dokaneheifard S., Mohammadi M. (2024). Global prevalence of polycystic ovary syndrome in women worldwide: A comprehensive systematic review and meta-analysis. Arch. Gynecol. Obstet..

[136] Gautam R., Maan P., Jyoti A., Kumar A., Malhotra N., Arora T. (2025). The role of lifestyle interventions in PCOS management: A systematic review. Nutrients.

[137] Alesi S., Forslund M., Melin J., Romualdi D., Peña A., Tay C.T., Witchel S.F., Teede H., Mousa A. (2023). Efficacy and safety of anti-androgens in the management of polycystic ovary syndrome: A systematic review and meta-analysis of randomised controlled trials. eClinicalMedicine.

[138] Balen A.H., Morley L.C., Misso M., Franks S., Legro R.S., Wijeyaratne C.N., Stener-Victorin E., Fauser B.C., Norman R.J., Teede H. (2016). The management of anovulatory infertility in women with polycystic ovary syndrome: An analysis of the evidence to support the development of global WHO guidance. Hum. Reprod. Update.

[139] Caruso G., Weroha S.J., Cliby W. (2025). Ovarian cancer: A review. JAMA.

[140] Cho K.R., Shih I.-M. (2009). Ovarian cancer. Annu. Rev. Pathol. Mech. Dis..

[141] NCI Cancer Stat Facts: Ovarian Cancer. https://seer.cancer.gov/statfacts/html/ovary.html.

[142] Kroeger P.T., Drapkin R. (2017). Pathogenesis and heterogeneity of ovarian cancer. Curr. Opin. Obstet. Gynecol..

[143] Abd Ghani M.F., Othman R., Nordin N. (2020). Molecular docking study of naturally derived flavonoids with antiapoptotic BCL-2 and BCL-XL proteins toward ovarian cancer treatment. J. Pharm. Bioallied Sci..

[144] Tang J., Zhuang Y., Zhang Y., Hu H., Wang H., Xu H., Li Y., Tu C. (2025). Necroptosis in cancer: Insight from epigenetic, post-transcriptional and post-translational modifications. J. Hematol. Oncol..

[145] He L., Peng K., Liu Y., Xiong J., Zhu F.-F. (2013). Low expression of mixed lineage kinase domain-like protein is associated with poor prognosis in ovarian cancer patients. OncoTargets Ther..

[146] Slaughter K., Holman L.L., Thomas E.L., Gunderson C.C., Lauer J.K., Ding K., McMeekin D.S., Moore K.M. (2016). Primary and acquired platinum-resistance among women with high grade serous ovarian cancer. Gynecol. Oncol..

[147] Walboomers J.M., Jacobs M.V., Manos M.M., Bosch F.X., Kummer J.A., Shah K.V., Snijders P.J., Peto J., Meijer C.J., Muñoz N. (1999). Human papillomavirus is a necessary cause of invasive cervical cancer worldwide. J. Pathol..

[148] Bowden S.J., Doulgeraki T., Bouras E., Markozannes G., Athanasiou A., Grout-Smith H., Kechagias K.S., Ellis L.B., Zuber V., Chadeau-Hyam M. (2023). Risk factors for human papillomavirus infection, cervical intraepithelial neoplasia and cervical cancer: An umbrella review and follow-up Mendelian randomisation studies. BMC Med..

[149] Cheng X., Wang P., Cheng L., Zhao F., Liu J. (2025). Trends in cervical cancer incidence and mortality in the United States, 1975–2018: A population-based study. Front. Med..

[150] Francoeur A.A., Monk B.J., Tewari K.S. (2025). Treatment advances across the cervical cancer spectrum. Nat. Rev. Clin. Oncol..

[151] NCI Cervical Cancer Prognosis and Survival Rates. https://www.cancer.gov/types/cervical/survival.

[152] Daneri-Navarro A., Macias-Lopez G., Oceguera-Villanueva A., Del Toro-Arreola S., Bravo-Cuellar A., Perez-Montfort R., Orbach-Arbouys S. (1998). Urokinase-type plasminogen activator and plasminogen activator inhibitors (PAI-1 and PAI-2) in extracts of invasive cervical carcinoma and precursor lesions. Eur. J. Cancer.

[153] Sugimura M., Kobayashi H., Kanayama N., Terao T. (1992). Clinical significance of urokinase-type plasminogen activator (uPA) in invasive cervical cancer of the uterus. Gynecol. Oncol..

